# Hospitalisation, mortality and years of life lost among chikungunya and dengue cases in Brazil: a nationwide cohort study, 2015–2024

**DOI:** 10.1016/j.lana.2025.101177

**Published:** 2025-07-07

**Authors:** Thiago Cerqueira-Silva, Luciana L. Cardim, Enny Paixão, Marta Rossi, Andreia Costa Santos, André Portela F de Souza, Gervasio Santos, Mauricio L. Barreto, Elizabeth B. Brickley, Julia M. Pescarini

**Affiliations:** a Faculty of Epidemiology and Population Health, London School of Hygiene & Tropical Medicine, London, UK; b Laboratório de Medicina e Saúde Pública de Precisão, Fundação Oswaldo Cruz, Salvador, Bahia, Brazil; c Centro de Integração de Dados e Conhecimento para a Saúde (CIDACS), Fundação Oswaldo Cruz, Salvador, Bahia, Brazil; d Faculty of Sports, Technology and Health Sciences, St Mary’s University, London, UK; e Fundação Getúlio Vargas, São Paulo, São Paulo, Brazil

**Keywords:** Dengue, Chikungunya, Competing risks, Prognosis, Latin America

## Abstract

**Background:**

The incidence of infections from arthropod-borne viruses, including chikungunya and dengue, is increasing globally. We used nationwide data collected over a decade in Brazil to examine the factors associated with hospitalisation, in-hospital mortality, and the years of life lost from these diseases in Brazil.

**Methods:**

Using nationwide de-identified chikungunya and dengue disease records registered from 1st January 2015 to 31 December 2024, we estimated the risk factors for hospitalisation and in-hospital mortality via logistic regression and the Fine and Gray model, respectively. We also calculated the years of life lost for each disease and the average of years of life lost (aYLL), stratified by geographic region, sex and race/ethnicity.

**Findings:**

We studied 1,125,209 chikungunya cases: 21,336 (1.9%) required hospitalisations. Among hospitalised cases, 1044 (4.9%) deaths occurred within 84 days of symptom onset, of which 728 (69.7%) were attributed to chikungunya. We studied 13,741,408 dengue cases: 455,899 (3.3%) required hospitalisation, with 12,969 (2.8%) deaths among the hospitalised cases, with 9989 (77.0%) attributed to dengue. Age (<1 or ≥70 years), sex (male), and the presence of diabetes and kidney disease were risk factors for hospitalisation and in-hospital mortality in both diseases. The aYLL for chikungunya was 16.0 years, and for dengue, 14.5 years; however, the burden was not evenly distributed across the population. For chikungunya, Black participants experienced the highest aYLL of 22.0 years, while White participants were the least affected (aYLL: 13.0). For dengue, the most affected group was Indigenous (aYLL: 22.5) and the least White (aYLL: 12.6).

**Interpretation:**

Infants, older people (≥70 years), male sex and the presence of comorbidities are associated with increased severity in cases of chikungunya and dengue. These diseases disproportionately affect historically minoritised populations, with participants who self-identified as Black and Indigenous experiencing significantly greater years of life lost compared to the white population. Mitigating the impacts of chikungunya and dengue necessitates addressing health and social inequities.

**Funding:**

10.13039/501100000288Royal Society, 10.13039/100010269Wellcome Trust, 10.13039/501100003593CNPq.

**Research in context**

**Evidence before this study**

We searched for published articles up to 12 December 2024, using the terms (chikungunya OR dengue) AND (“years of life lost” OR burden) OR (hospitalisation risk factors) OR (mortality risk factors), without language restrictions. We identified 804 papers. After screening titles and abstracts, we noted that, despite multiple studies evaluating mortality risk factors for chikungunya and dengue cases, the studies utilising Brazil’s national notification system suffer from one or more methodological flaws, including dichotomising age, ignoring competing events, selecting variables based on p-values, or employing stepwise procedures. Furthermore, studies assessing years of life lost did not disaggregate data by race or ethnicity, only presenting the overall value for Brazil.

Considering the global spread of dengue and chikungunya and the availability of high-quality surveillance data from Brazil, we investigated the risk factors associated with hospitalisation and in-hospital mortality in patients with these diseases. We used a harmonised approach to evaluate the risk factors for complications in both diseases. Additionally, we assessed years of life lost, disaggregated by race/ethnicity and geographic region, to quantify the differences in health inequalities related to the burden of chikungunya and dengue.

**Added value of this study**

We found that male sex, age extremes (either younger than one year or older than 80 years), and the presence of comorbidities (e.g., diabetes and kidney diseases) are risk factors for hospitalisation and in-hospital mortality in patients with chikungunya or dengue. Importantly, we demonstrate that the burden of these diseases is disproportionately high among marginalised populations. Hospitalised chikungunya cases who self-identified as Black exhibited the highest average years of life lost, whereas, for dengue, this burden was greatest among Indigenous individuals, who experienced at least 50% more than their White counterparts.

When analysed by geographic region, individuals in the North and Northeast regions exhibit a higher average of years of life lost than those from the Southeast. These patterns align with findings showing that municipalities with higher proportions of Black and Indigenous populations experienced deaths at younger ages due to these diseases. These results highlight significant health inequalities based on race/ethnicity and geographic region in the burden caused by chikungunya and dengue.

**Implications of all the available evidence**

The identification of risk factors for mortality related to both chikungunya and dengue, such as male sex, very young and very old ages, and the presence of comorbidities, can inform clinical and public health decisions, including improved access to healthcare for vulnerable groups and prioritisation of preventive efforts in regions with a high concentration of at-risk populations. The disproportionate impact of chikungunya and dengue on marginalised groups highlights the significant effects of systemic racism and adverse living conditions. Limited access to healthcare, lower socioeconomic status, and other structural inequities amplify the vulnerability of these populations to severe disease outcomes, including death at younger ages. Addressing structural and systemic inequalities will be essential for reducing the adverse consequences of chikungunya and dengue and mitigating their disproportionate burden on marginalised populations.

## Introduction

In recent decades, there has been a global surge in the incidence arthropod-borne virus (arbovirus) infections, such as Chikungunya (CHIKV) and Dengue (DENV), likely due to viral evolution, changing land use, vector expansion, increasing urbanisation, human mobility, and climate change.^1^ In 2023, more than 500,000 chikungunya cases and more than 6.5 million dengue cases were reported worldwide. The numbers rose further in 2024; when there were over 480,000 chikungunya cases, resulting in 190 chikungunya-related deaths and over 14 million dengue cases, resulting in 10,000 dengue-related deaths.^2^^,^^3^ Brazil is among the countries with the highest burden of arboviruses, accounting for more than 70% of all notified dengue and chikungunya cases worldwide in 2024.^4^^,^^5^

CHIKV and DENV infections are typically asymptomatic or mild and are considered self-limiting diseases with low case fatality rates, according to World Health Organization official guidelines. However, multiple studies have demonstrated chikungunya as an important cause of death. Several studies from Brazil, Jamaica, India, and the Dominican Republic have documented excess mortality during chikungunya outbreaks—findings that diverge from official death records attributed to the infection.^6^, ^7^, ^8^ Moreover, a population-based cohort study identified a heightened risk of death within three months post-infection, particularly among men and older adults.^9^ Finally, pathophysiological research further supports the biological plausibility of this association.^10^

Furthermore, some patients may suffer from chronic joint pain following chikungunya, reducing their quality of life.^11^ Similarly, during the acute phase, dengue can progress to severe dengue, characterised by complications such as plasma leakage, severe bleeding, and/or organ failure.^12^^,^^13^ These acute complications of severe dengue both increase the immediate risk of death and also carry the potential to cause long-term sequelae, including persistent fatigue and joint and muscle pain.^14^

Vector control for the *Aedes* spp. mosquitoes, which transmit CHIKV and DENV, is challenging because they are highly adaptable and capable of breeding in almost any water source, and many vector control measures (e.g., insecticides) have limited effectiveness and recurrent costs.^15^ The use of Wolbachia-infected mosquitoes has been successful^16^; however, comprehensive studies on the environmental impact of this approach still needs to be conducted. Safe and effective vaccines present a complementary solution for reducing risks from arbovirus infections.^17^ As of 2023, CHIKV and DENV have licensed vaccines, albeit with limited availability and restrictions regarding eligible age ranges which can hamper the applicability in endemic settings.^18^ Chikungunya and dengue lack clinically approved antiviral therapeutics and depend solely on supportive clinical management.^19^^,^^20^ Identifying people at high risk for severe illness or mortality from chikungunya or dengue is essential for informing public health initiatives and optimising healthcare resource allocation.

While previous studies have examined the risk factors associated with severe outcomes for dengue and chikungunya cases,^21^, ^22^, ^23^, ^24^, ^25^ many have been underpowered to evaluate the risk factors for mortality.^21^, ^22^, ^23^, ^24^ The largest previous study on chikungunya-related mortality assessed 182,731 cases, resulting in 383 deaths, and identified comorbidities, such as diabetes, hypertension and chronic kidney disease, as risk factors.^24^ The largest previous study of dengue assessed 678,836 cases, resulting in 3225 deaths, and identified comorbidities, such as pulmonary disease and ischaemic heart disease, as risk factors for in-hospital mortality.^25^

The current study aims to use nationwide data collected over a decade in Brazil to identify demographic and clinical risk factors associated with hospitalisation and in-hospital mortality among patients registered with chikungunya and dengue. To mitigate bias, the current study will use a competing risk analysis to differentiate between deaths attributable to chikungunya or dengue and deaths from other causes. Additionally, to better reflect the health disparities by sex, racial/ethnic group, and region, this study will quantify the years of life lost for each disease within these subgroups.

## Methods

### Data sources

We conducted a cohort study using data on confirmed cases of chikungunya and dengue from the Brazilian National Notifiable Disease Information System (*Sistema de Informação de Agravos de Notificação*–SINAN), which mandates reporting of all suspected chikungunya and dengue cases at public and private health facilities based on the attending clinician’s initial clinical diagnosis (i.e., not laboratory-confirmed). SINAN uses standardised forms to collect data on the patients’ clinical and sociodemographic characteristics and outcomes, including hospitalisation and death (classified as death from chikungunya/dengue or death from other causes). All cases in SINAN are classified retrospectively following an epidemiological investigation and are considered confirmed with (i) laboratory evidence (i.e., enzyme-linked immunosorbent assay or reverse transcription polymerase chain reaction) or (ii) clinical-epidemiological criteria (i.e., symptomatology consistent with the disease occurring in the same area and time with a high incidence of other confirmed cases).

We estimated life expectancy using the information on population size (i.e., by age, sex, state and race/ethnicity) from the 2022 Brazilian census and deaths (i.e., by age, sex, state and race/ethnicity) from the 2022 Brazilian Mortality Information System (*Sistema de Informação sobre Mortalidade* -SIM).

### Study population and variables

Our study population included every person registered in SINAN with a confirmed episode of chikungunya or dengue between January 01, 2015, and December 31, 2024. From each registration, we extracted information about the individual’s demographics (sex, region of residence, race/ethnicity and education level), self-reported symptoms, comorbidities, dates of symptom onset, date of hospital admission, date of death and cause of death (due to chikungunya, dengue or other causes).

### Outcomes and covariates

The primary outcomes were hospitalisation and in-hospital mortality.

The covariates included age (≤1, 1–9, 10–19, 20–39–as the reference level, 40–49, 50–59, 60–69, 70–79, 80–89, >90 years), sex, presence of comorbidities (diabetes, liver disease, hypertension, autoimmune diseases, haematological diseases, chronic kidney diseases), year of symptoms onset and geographic region (North, Northeast, Southeast, South, and Central-west). For comorbidities, we considered missing values as having “no comorbidities”, as previously described.^26^ We evaluated deaths within 84 days (12 weeks) of symptom onset, as we previously reported an increased risk of death due to chikungunya during this period.^9^ Because the SINAN database only records the hospital admission date, we defined in-hospital mortality as death occurring among participants with both hospitalisation and death records.

### Statistical analysis

To investigate the distribution of chikungunya and dengue cases across the country, we calculated the incidence per state for each year of the study period. We used logistic regression adjusted for sex, age, comorbidities, geographic region and year of diagnosis to assess the risk factors for hospitalisation. We estimated the cumulative incidence function (CIF) by cause of death, stratified by the presence of hospitalisation records. To account for the competing risk (death by chikungunya, dengue or other causes), we then used the Fine and Gray model, with days since symptom onset as the timescale, to assess the risk factors for death due to chikungunya or dengue (Supplementary Methods).^27^ Participants were followed since symptom onset up to 84 days post-symptom onset. The model was adjusted for the same variables as the hospitalisation model. In the chikungunya models, dengue-related deaths were classified as other causes, while chikungunya-related deaths were similarly classified as other causes in the dengue models.

We then calculated the life expectancy stratified by sex, geographic region of residence (or State of residence, if ≥10 deaths per State), and race/ethnicity. Using these estimates, we calculated the years of life lost due to premature death (years of life lost—YLL and average years of life lost–aYLL), using the age of death (due to chikungunya/dengue or other causes) recorded in SINAN, stratified by the same variables. We used the approach proposed by the Global Burden of Disease Study for YLL estimation with a discount rate of 0.03 (3%) (Supplementary Methods).^28^ The YLL compare the age at death with the life expectancy, providing interpretable estimates of the impact of premature mortality from specific causes of death. The Global Burden of Disease approach also considers that deaths in the productive years have a greater burden than those in the very young or very old, making it less sensitive to deaths occurring in the extreme ages. The aYLL allows for comparing the burden across subgroups by averaging the YLL based on the number of deaths in each group.^29^ The YLL was calculated for each 10-year interval (<10, 10–19, 20–29, 30–39, 40–49, 50–59, 60–69, 70–79, ≥80 years) and summed to derive the total YLL per group. All calculations were done for all-cause inpatient deaths.

Finally, we conducted an exploratory analysis to investigate the association between the proportion of people from minoritised backgrounds (Black, Indigenous and Mixed) by the municipality and the average age of death for all inpatient cases. We determined the relative index of inequality (RII) and the slope index of inequality (SII). While the RII measures relative disparities, the SII assesses absolute disparities.^30^ Both absolute and relative measures of inequality were estimated using linear regression. We used the population by race/ethnicity per municipality from the 2022 Brazilian census, which collects self-reported data. The proportion of minorities was categorised into quintiles.

### Sensitivity analysis

We also conducted several sensitivity analyses. First, to test the susceptibility of the results to misclassification of the exposure, we conducted a sensitivity analysis for chikungunya and dengue using only laboratory-confirmed cases. Second, we assessed the possible bias caused by the disruption of the healthcare system due to the COVID-19 pandemic by evaluating the risk factors for hospitalisation, inpatient death, and years of life lost in three periods: 2015–2019, 2020–2022, and 2023–2024.

All analyses were performed in R, version 4.4.1, using the following packages: tidyverse, tidycmprsk, and yll.

### Ethics

This study was approved by the Ethical Committee of Oswaldo Cruz Foundation (4.756.567) and the London School of Hygiene & Tropical Medicine (25339/RR/24583).

### Role of funding source

The study’s funders had no role in the design, data collection, data analysis, data interpretation, or writing of the report.

## Results

### Chikungunya and dengue cases in Brazil

Between 1 January 2015 and 31 December 2024, 1,125,209 chikungunya cases were reported by the Brazilian Ministry of Health (SINAN). All 27 states of Brazil reported cases during this period, with the State of Minas Gerais in the Southeast region reporting the most cases (278,033). The year 2024 recorded the highest number of cases (216,910) in Brazil, followed by 2017 (165,158) (Supplementary Fig. S2). A total of 21,336 (1.9%) of the chikungunya cases required hospitalisation. Within 84 days of chikungunya symptom onset, 4.9% (1044/21,336) patients died, and 728 (69.7%) of those deaths were attributed to chikungunya (Table 1, Supplementary Fig. S3).

**Table 1:** Characteristics of study participants.

Characteristic	Chikungunya		Dengue	
	Inpatient	Outpatient	Inpatient	Outpatient
	N = 21,336	N = 1,103,873	N = 455,899	N = 13,285,509
Age–years, median (IQR)	34 (13, 57)	39 (24, 54)	38 (19, 59)	33 (20, 49)
**Age group in years**				
<1	1037 (4.9)	9079 (0.8)	9393 (2.1)	116,966 (0.9)
1–9	3050 (14.3)	56,565 (5.1)	46,957 (10.3)	993,722 (7.5)
10–19	2679 (12.6)	122,644 (11.1)	63,450 (13.9)	2,091,513 (15.7)
20–39	5240 (24.6)	372,327 (33.7)	118,476 (26.0)	4,815,079 (36.2)
40–49	2412 (11.3)	188,405 (17.1)	55,934 (12.3)	2,003,314 (15.1)
50–59	2225 (10.4)	160,852 (14.6)	51,437 (11.3)	1,576,089 (11.9)
60–69	1926 (9.0)	112,057 (10.2)	45,930 (10.1)	1,025,632 (7.7)
70–79	1548 (7.3)	58,804 (5.3)	37,538 (8.2)	490,145 (3.7)
80–89	997 (4.7)	20,272 (1.8)	21,983 (4.8)	152,903 (1.2)
≥90	222 (1.0)	2868 (0.3)	4801 (1.1)	20,146 (0.2)
Sex–Female	12,106 (56.7)	679,158 (61.5)	245,677 (53.9)	7,294,453 (54.9)
**Geographic region**				
North	1762 (8.3)	38,108 (3.5)	18,440 (4.0)	270,636 (2.0)
Northeast	10,473 (49.1)	554,746 (50.3)	67,312 (14.8)	1,231,529 (9.3)
Southeast	7394 (34.7)	447,579 (40.5)	187,728 (41.2)	7,759,783 (58.4)
South	219 (1.0)	3141 (0.3)	75,762 (16.6)	2,161,362 (16.3)
Central west	1488 (7.0)	60,299 (5.5)	106,615 (23.4)	1,861,809 (14.0)
Missing	–	–	42 (<0.1)	390 (<0.1)
**Education level**				
Elementary school <5 years of schooling	1755 (8.2)	58,909 (5.3)	37,176 (8.2)	706,776 (5.3)
Elementary school 6–9 years of schooling	2171 (10.2)	91,657 (8.3)	47,362 (10.4)	1,242,178 (9.3)
High School	3448 (16.2)	161,839 (14.7)	83,145 (18.2)	2,762,921 (20.8)
College	1216 (5.7)	42,908 (3.9)	30,138 (6.6)	727,592 (5.5)
Missing	12,746 (59.7)	748,560 (67.8)	258,078 (56.6)	7,846,042 (59.1)
**Race/Ethnicity**				
Asian	125 (0.6)	10,477 (0.9)	4722 (1.0)	109,625 (0.8)
Black	915 (4.3)	49,626 (4.5)	15,358 (3.4)	552,536 (4.2)
Indigenous	69 (0.3)	3047 (0.3)	1272 (0.3)	29,217 (0.2)
Mixed	13,109 (61.4)	604,003 (54.7)	172,623 (37.9)	4,369,833 (32.9)
White	4429 (20.8)	159,491 (14.4)	193,254 (42.4)	5,322,305 (40.1)
Missing	2689 (12.6)	277,229 (25.1)	68,670 (15.1)	2,901,993 (21.8)
Diabetes	1730 (8.1)	39,842 (3.6)	34,411 (7.5)	415,327 (3.1)
Auto immune disease	304 (1.4)	5237 (0.5)	5550 (1.2)	68,116 (0.5)
Hematologic disease	227 (1.1)	4128 (0.4)	5007 (1.1)	58,295 (0.4)
Liver disease	258 (1.2)	4465 (0.4)	4536 (1.0)	59,220 (0.4)
Hypertension	3563 (16.7)	98,956 (9.0)	70,709 (15.5)	989,710 (7.4)
Kidney disease	353 (1.7)	3954 (0.4)	6426 (1.4)	51,651 (0.4)
Time between symptom onset and hospitalisation—days, median (IQR)	3 (1–6)	–	4 (2–6)	–
**Confirmation criteria**				
Laboratory	12,243 (57.4)	386,880 (35.1)	266,987 (58.6)	5,008,330 (37.7)
Clinical epidemiological	9093 (42.6)	716,993 (64.9)	188,912 (41.4)	8,277,179 (62.3)
**Outcome**				
Cure	20,292 (95.1)	1,103,165 (>99.9)	442,930 (97.2)	13,282,600 (>99.9)
Death chikungunya or dengue^a^	728 (3.4)	511 (<0.1)	9989 (2.2)	2093 (<0.1)
Death other causes	316 (1.5)	197 (<0.1)	2980 (0.7)	816 (<0.1)

a In the chikungunya cases, dengue-related deaths were classified as other causes, while chikungunya-related deaths were similarly classified as other causes in the dengue cases.

During the same period, 13,741,408 dengue cases were reported in SINAN. All 27 states reported cases, with the State of São Paulo in the Southeast region reporting the highest number of cases (4,434,470). Similar to chikungunya, 2024 recorded the highest number of dengue cases in Brazil, with 5,827,068 cases (Supplementary Fig. S5). A total of 455,899 (3.3%) of the dengue cases required hospitalisation. Within 84 days of dengue symptom onset, 2.9% (12,969/455,899) patients died, and 9989 (77.0%) of these deaths were attributed to dengue (Table 1, Supplementary Fig. S6).

### Clinical and demographic risk factors for hospitalisation and mortality

**Fig. 1:**
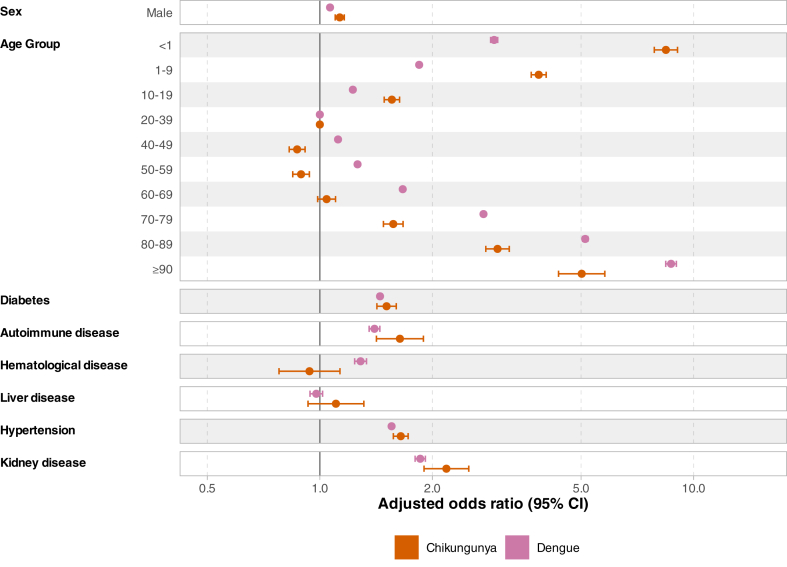
**Risk factors for hospitalisation among chikungunya and dengue cases.** Lines represent 95% confidence intervals. The models were also adjusted for the year of symptom onset and geographic region.

**Fig. 2:**
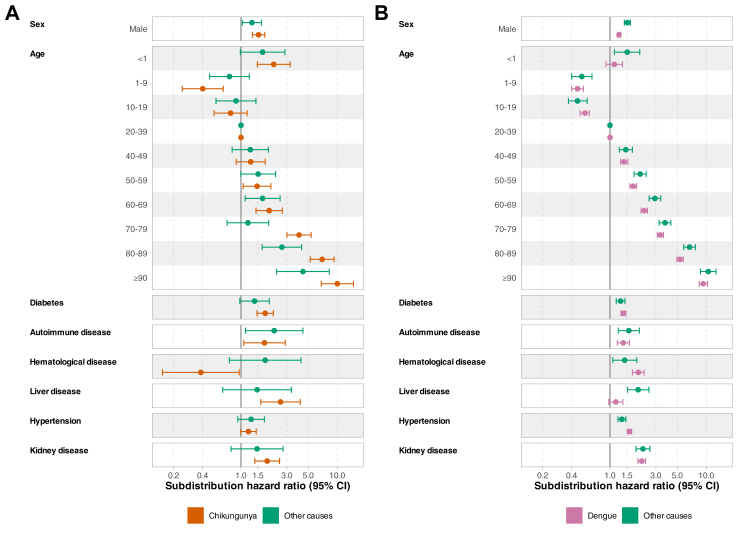
**Risk factors for inpatient death among (A) chikungunya and (B) dengue cases.** Lines represent 95% confidence intervals. The models were also adjusted for the year of symptom onset and geographic region.

For chikungunya, we found that the oldest and youngest age groups (<20 and ≥70 years), male sex, and the presence of specific comorbidities (i.e., diabetes, autoimmune diseases, hypertension, and kidney disease) were risk factors for hospitalisation (Fig. 1, Supplementary Table S1). The cumulative incidence function (CIF) of death shows a similar pattern in the timing of death among inpatients and outpatients, with close to 90% of the deaths occurring in the first 49 days and plateauing afterwards (Supplementary Fig. S4). However, the outpatient group has a very low proportion of deaths (708–<0.05%) (Supplementary Fig. S4). Among hospitalised patients, age (<1 and ≥60 years), male sex, diabetes, kidney and liver disease were risk factors for death due to chikungunya. Regarding death due to other causes, autoimmune disease was also a risk factor (Fig. 2, Supplementary Table S2).

For dengue, we also found that age extremes (<20 and ≥60 years), male sex, and the presence of specific comorbidities (i.e., diabetes, autoimmune diseases, hypertension, haematological and kidney disease) are risk factors for hospitalisation (Fig. 1, Supplementary Table S1). The CIF for death was very similar to that for chikungunya, but with an earlier plateau around 35 days post-symptom onset (Supplementary Fig. S7). Among hospitalised patients, those aged 1–19 years had a lower risk of death compared to participants aged 20–39 years; however, the risk of death increased incrementally with each additional decade of age beyond 40 years. Also, male sex and the presence of specific comorbidities (i.e., diabetes, hypertension, kidney, and autoimmune diseases) were risk factors for death due to dengue and death due to other causes.

### Years of lives lost (YLL) from chikungunya and dengue

**Fig. 3:**
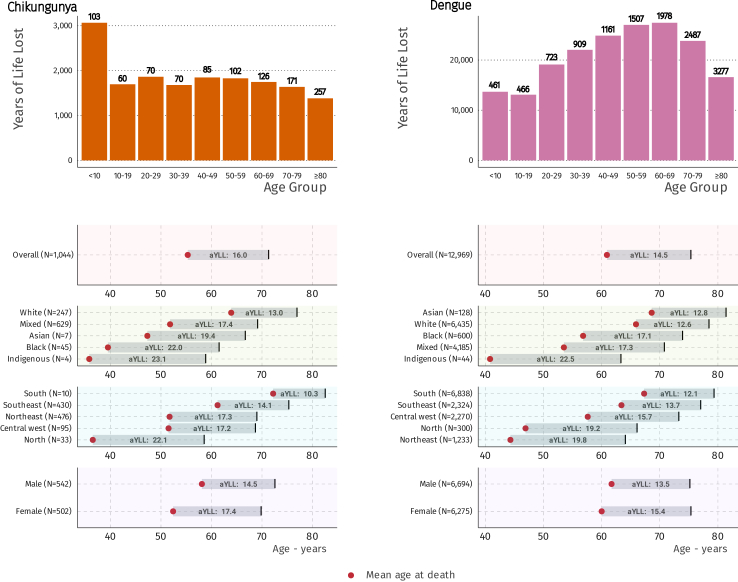
**Top panel: Years of Life lost per age interval of in-hospital death from any cause for chikungunya and dengue.** Numbers in the histogram indicate the size of each group. **Bottom panel: average years of life lost (aYLL) per group.** Numbers in parentheses indicate the size of each group; estimates are stratified by geographic region, race/ethnicity, and sex. Red dots represent the mean age at death. The analyses are based on the complete case analyses (race/ethnicity missing data: chikungunya: 112-10.7%, dengue: 1577–12.2%, geographic region: dengue: 4-0.03%).

The age group with the highest YLL from chikungunya was those aged <10 years, while the other age groups exhibited similar levels of YLL. The mean age at death, from any cause, among hospitalised chikungunya cases was 55.3 years, resulting in an average YLL (aYLL) of 16.0 years. Subgroup analysis revealed notable differences by sex, race/ethnicity, and geographic region, with an average age at death following chikungunya of 52.4 years (aYLL—17.4) for women and 58.1 years (aYLL–14.5) for men, respectively. We observed both racial and geographic disparities, with the most affected groups including participants identifying as Black, with a mean age at death of 39.5 years (aYLL–22.0), and participants residing in the North region, with a mean age at death of 36.5 years (aYLL–22.1) (Fig. 3, Supplementary Table S3).

**Fig. 4:**
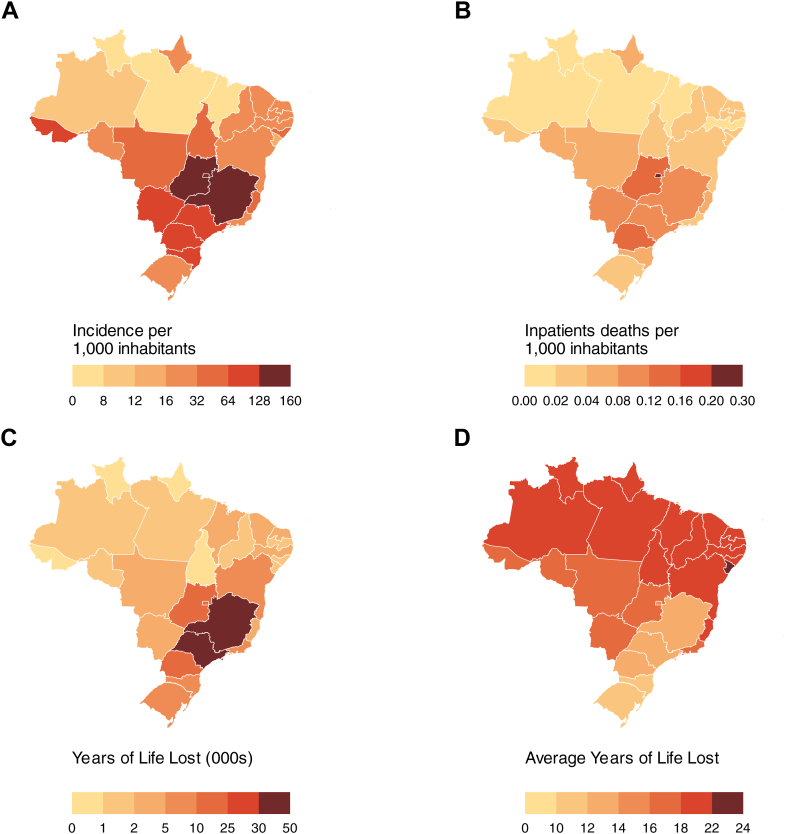
**Distribution of dengue cases and deaths between January 2015 and June 2024. (A)** Cumulative dengue incidence per state by 1000 inhabitants. **(B)** Cumulative inpatient deaths of dengue cases attributable to dengue and other causes per state by 1000 inhabitants. **(C)** Years of life lost among inpatients deaths attributable to dengue and other causes. **(D)** Average years of life lost among inpatient deaths attributable to dengue and other causes.

The age group with the highest YLL from dengue was 60–69 years, with similar figures for those aged 50–59. The mean age at death, from any cause, among hospitalised dengue cases was 60.9 years, resulting in an aYLL of 14.5. Subgroup analysis indicated similar patterns to chikungunya by sex, with a mean age at death following dengue of 60.0 years (aYLL-15.4) for women and 61.7 years (aYLL-13.5) for men. However, the patterns varied by race/ethnicity and geographic region, with the most affected groups including participants identifying as Indigenous, who had a mean age at death of 40.8 years (aYLL–22.5), and participants residing in the Northeast region, with a mean age at death of 44.3 years (aYLL–19.8) (Fig. 3, Supplementary Table S5). The analysis by state indicates that the Southeast and South regions exhibit the highest cumulative incidence and inpatient mortality rates of dengue per 1000 inhabitants over the study period, yet, had lower average years of life lost than the other regions (Fig. 4).

### Sensitivity analysis

The sensitivity analysis using only laboratory-confirmed cases for chikungunya and dengue yielded consistent results with the main analysis but with wider confidence intervals (Supplementary Tables S6–S8). A similar pattern was seen with the analysis stratified relative to COVID-19: pre (2015–2019), during (2020–2022), and post (2023–2024), consistent results with wider confidence intervals (Supplementary Tables S9 and S10).

Finally, evaluating the SII and RII for the age of death of inpatient cases regarding the proportion of minorities in each city revealed pronounced inequality. For chikungunya cases, the SII was 24.1 (95% CI 23.2–33.0) and the RII was 1.52 (95% CI 1.29–1.75). In the case of dengue, the SII was 27.7 (95% CI 26.0–29.4), and the RII was 1.62 (95% CI 1.57–1.68) (Supplementary Table S11). This indicates that in municipalities with a lower proportion of minorities, the average age of death for inpatient cases is higher for both diseases.

## Discussion

In this study, we analysed data from Brazil, the country with the highest number of chikungunya and dengue cases. We replicated previous findings^22^^,^^24^^,^^25^^,^^31^^,^^32^ that male sex, older age and presence of comorbidities are risk factors for hospitalisation and mortality in cases of chikungunya or dengue. Our results also show that the average years of life lost due to chikungunya or dengue varies by sex, race/ethnicity, and geographic region. Both chikungunya and dengue led to more years of life lost in the Northeast and North regions, where there are the highest levels of community-level and individual-level poverty in Brazil.^33^ When analysed by race/ethnicity, participants who self-identified as Black, Mixed or Indigenous populations experienced the highest aYLL, with a greater than 50% increase in the years of life expectancy that were lost compared to participants identifying as White.

Our study found that participants with comorbidities were at increased risk of hospitalisation and in-hospital mortality following chikungunya and dengue. These findings align with those of previous studies using the SINAN database, which showed that older age (≥60 for chikungunya and ≥55 years for dengue) and the presence of comorbidities (chronic kidney disease, diabetes, hypertension) were associated with increased chikungunya and dengue mortality.^22^^,^^24^^,^^25^^,^^31^^,^^32^ Overall, our study advances on previous studies by: (i) not selecting variables for the risk-factor model using a univariate p-value to decide which covariate to include, which can lead to a selection of non-confounders, mediators, or variables without public health significance and resulting in artificially small p-values and narrow confidence intervals^34^; (ii) not dichotomising age, which missed the increased risk of complications in children under 1 year of age,^34^ and (iii) not modelling death using logistic regression, which doesn’t account for the timing of death or the competing risk of death due to other causes; this can bias the results in the presence of common risk factors for both causes, such as comorbidities, creating spurious protective or harmful associations depending on the association of the risk factor with the competing event.^27^

Our study differs from past studies by modelling death as a survival analysis, applying a competing risk approach for identifying risk factors for death, and consistently using the same variables in the models for hospitalisation and death across both diseases, facilitating comparability. Secondly, we restricted the analysis of risk factors for mortality to inpatient cases, as the proportion of deaths among the outpatient cases was too low (<0.05%) and may be biased towards representing a particularly vulnerable population. Third, we used data from the 2022 census and corresponding mortality data, which provided a more up-to-date representation of the Brazilian population to estimate YLL. Finally, we provided multiple sources of evidence for each question evaluated (e.g., exposure: analysis of only laboratory-confirmed cases, follow-up period: stratification by COVID-19 timing); the consistency of the results reinforced the robustness of our findings. Considering the large sample size and diversity of the Brazilian context, our results are likely generalisable to other populations, particularly those in lower-income and middle-income countries with demographic profiles similar to Brazil.

Research from Brazil and other regions consistently demonstrate excess mortality during chikungunya outbreaks.^6^, ^7^, ^8^ In our study, chikungunya cases showed a death rate of 3.4%, compared to dengue cases (2.2%), highlighting differences in disease severity. Misclassification of chikungunya as dengue—due to similar symptoms, limited knowledge among healthcare workers and low specificity of serological tests due to cross-reactivity between dengue and chikungunya^35^—can delay the detection of chikungunya outbreaks, affecting timely resource use and proper clinical protocols. Past research has shown that suspected arbovirus cases are often reported as dengue rather than chikungunya.^36^ This scenario stresses the need for better training and awareness among healthcare workers to improve the accurate identification and management of chikungunya.

We found that older adults are especially vulnerable to severe disease from both infections, with this pattern particularly pronounced for dengue. This finding is critical given that the approved vaccines for dengue were tested only in participants aged 4 to 60.^37^ The Strategic Advisory Group of Experts on Immunization guidelines recommend vaccination primarily for those aged 6 to 16.^38^ However, the elevated risk of death in older populations underscores the need to reconsider vaccination strategies to protect this vulnerable age group better, including conducting effectiveness studies to assess the degree of protection from severe outcomes for participants not included in the existing clinical trials.

Although in our study, older people and those with comorbidities were, on average, at the highest risk of death by chikungunya and dengue, those diseases pose a higher burden on premature mortality to minoritised groups. Among the hospitalised cases, the mean age at death for chikungunya was 55 years, 5 years younger than those dying by dengue, with an aYLL of 16.0 for chikungunya and 14.5 for dengue. However, the highest values of aYLL were observed among Black and Indigenous populations and people living in Brazil’s Northern and Northeastern regions. Systemic racism, reflecting Brazil’s colonial legacies, drastically affects the health and well-being of Black and mixed-race communities today.^33^ Relative to their white counterparts, Black and mixed-race populations experience poorer socioeconomic positions, manifesting in precarious housing conditions, lower access to basic sanitation, lower incomes, and lower access to healthcare services.^33^^,^^39^^,^^40^

These structural inequalities have exacerbated the impact of neglected tropical diseases on communities, resulting in poorer health outcomes and higher mortality rates.^41^ Systemic racism acts as a critical social determinant of health, directly affecting disease prevalence and perpetuating social and health inequities. Racial inequalities are reflected across geographical regions, with the North and Northeast having the highest proportion of Black and mixed-race residents, along with the highest poverty rates.^33^^,^^42^ In our study, we found a strong correlation between the mean age of death and an index measuring poverty levels and the proportions of Black, mixed-race, and Indigenous populations in municipalities. Municipalities with higher poverty and greater proportions of these populations had lower mean ages of death. The largest aYLL among ethnic and socially marginalised groups is also supported by the inequality measures (SII/RII) between the mean age of death and the proportion of Black, mixed-race, and Indigenous people in each municipality. Municipalities with higher proportions of these populations exhibited lower mean ages of death. This provides further evidence of the racialised burden of these diseases and highlights the importance of intersectoral efforts to address underlying social inequalities to improve health.

We must interpret our study estimates with caution. These estimates should be seen as prognostic factors rather than causal effects because we lack key confounders necessary to estimate causal effects, such as the data on previous infections and detailed socioeconomic information, including the healthcare quality provided to participants. In addition, our study period partially overlaps with the restrictions of the COVID-19 pandemic, which could distort the results. However, the analysis stratified by pre-COVID-19, COVID-19, and post-COVID-19 yielded consistent results but with wider confidence intervals.

Our study also has some additional limitations. Firstly, we did not have information about the specific causes of participants whose deaths were not due to chikungunya or dengue, as SINAN forms categorise all deaths not attributable to chikungunya/dengue as other causes. Secondly, only 40% of cases were confirmed through laboratory testing. This low proportion aligns with Brazilian guidelines, which prioritise laboratory confirmation until a predefined incidence threshold is reached, after which cases are diagnosed clinically. Thirdly, SINAN contains incomplete hospitalisation data compared to the hospitalisation information system.^43^ However, any underreporting would likely be non-differential, potentially biasing results toward the null. Fourthly, clinical similarities between chikungunya and dengue may result in misclassification between the two diseases. Nonetheless, restricting the analysis to laboratory-confirmed cases yielded similar findings. Fifthly, we had missing data (around 12%) for race/ethnicity, affecting our YLL analysis. However, the analysis evaluating the proportion of minorities (Black, Indigenous and Mixed) and the RII and SII of the mean age at death showed pronounced disparities in the same direction; municipalities with a higher proportion of minorities had a lower mean age at death.

### Conclusions

Our study offers precise estimates of risk factors for complications from chikungunya and dengue, which can guide clinical and public health decisions. Age extremes and comorbidities are strongly linked to a higher risk of hospitalisation and in-hospital death in patients with these diseases. However, chikungunya and dengue disproportionately burden marginalised populations, with Black and Indigenous populations and those in the North and Northeast regions experiencing significantly more years of life lost. Therefore, addressing these health and social inequities is essential for mitigating the impacts of chikungunya and dengue.

## Contributors

TCS, JMP and EBB conceptualised the study. TCS analysed the data. TCS and JMP contribute to data visualisation. TCS and JMP accessed and verified the raw data in the study. TCS wrote the first draft of the paper. EP, ACS, JMP, and EBB contributed to interpreting the results. LC, MR, ACS, APS, GS, MLB critically reviewed the manuscript. All authors contributed to manuscript editing and revision.

## Data sharing statement

All data used in this article is publicly available at: https://datasus.saude.gov.br/ and https://www.ibge.gov.br/.

## Editor’s note

The Lancet Group takes a neutral position with respect to territorial claims in published maps and institutional affiliations.

## Declaration of interests

The authors declared no conflicts of interest.

## Appendix A Supplementary data

Supplementary Figs. S1–S7 and Tables S1–11

Translated Summary
