# Supplementary material for: Hospitalisation, mortality and years of life lost among chikungunya and dengue cases in Brazil: a nationwide cohort study, 2015–2024
Source: Lancet Reg Health Am. 2025 Jul 7;49:101177. doi: 10.1016/j.lana.2025.101177 (PMC12273570; doi:10.1016/j.lana.2025.101177)
Supplement: Supplementary Figs. S1–S7 and Tables S1–11 [file mmc1.docx]

[Supplementary Table 1: Risk factors for hospitalisation for chikungunya and dengue. N refers to the number of cases in each subgroup. 3](#_Toc201566805)

[Supplementary Table 2: Risk factors for death after chikungunya estimated using Fine and Gray model. N refers to the number of cases in each subgroup; sHR = sub-distribution hazard ratio 5](#_Toc201566806)

[Supplementary Table 3 – Years of life lost (YLL) and average years of life lost (aYLL) for chikungunya inpatient deaths (all-cause) stratified by sex, geographic region and race/ethnicity. 7](#_Toc201566807)

[Supplementary Table 4: Risk factors for death after dengue estimated using Fine and Gray model. sHR = sub-distribution hazard ratio 8](#_Toc201566808)

[Supplementary Table 5 – Years of life lost (YLL) and average years of life lost (aYLL) for dengue inpatient deaths (all-cause) stratified by sex, geographic region and race/ethnicity. 10](#_Toc201566809)

[Supplementary Table 6: Risk factors for hospitalisation for chikungunya and dengue using only laboratory-confirmed cases. 10](#_Toc201566810)

[Supplementary Table 7: Risk factors for death following chikungunya estimated using Fine and Gray model, using only laboratory-confirmed cases. 12](#_Toc201566811)

[Supplementary Table 8: Risk factors for death following dengue estimated using Fine and Gray model, using only laboratory-confirmed cases. 14](#_Toc201566812)

[Supplementary Table 9: Risk factors for death following chikungunya estimated using Fine and Gray model stratified by period of COVID-19 pandemic. Pre-COVID (2015-2019), During COVID-19 (2020-2022) and Post COVID-19 (2023-2024). sHR = sub-distribution hazard ratio; CI = Confidence Interval 16](#_Toc201566813)

[Supplementary Table 10: Risk factors for death following dengue estimated using Fine and Gray model stratified by period of COVID-19 pandemic. Pre-COVID (2015-2019), During COVID-19 (2020-2022) and Post COVID-19 (2023-2024). sHR = sub distribution hazard ratio; CI = Confidence Interval 19](#_Toc201566814)

[Supplementary Table 11: Number of deaths by proportion of minorities by municipality 23](#_Toc201566815)

[Supplementary Figure 1: Flowchart of the study population from surveillance databases 24](#_Toc201566816)

[Supplementary Figure 2: Incidence per year of chikungunya by State in Brazil. 25](#_Toc201566817)

[Supplementary Figure 3: Histogram with the number of in-patient deaths after chikungunya by time since symptom onset stratified by the cause of death 26](#_Toc201566818)

[Supplementary Figure 4: Cumulative incidence function of death after chikungunya by time since symptom onset. 27](#_Toc201566819)

[Supplementary Figure 5: Incidence per year of dengue by State in Brazil. 28](#_Toc201566820)

[Supplementary Figure 6: Histogram with the number of in-patient deaths after dengue by time since symptom onset stratified by the cause of death 29](#_Toc201566821)

[Supplementary Figure 7: Cumulative incidence function of death after dengue by time since symptom onset. 30](#_Toc201566822)

[Appendix Methods 31](#_Toc201566823)

# Supplementary Table 1: Risk factors for hospitalisation for chikungunya and dengue. N refers to the number of cases in each subgroup.

|  | **Chikungunya** | | | | | **Dengue** | | | | |
| --- | --- | --- | --- | --- | --- | --- | --- | --- | --- | --- |
| **Characteristic** | **N** | **Event N** | **OR** | **95% CI** | **p-value** | **N** | **Event N** | **OR** | **95% CI** | **p-value** |
| **Year** |  |  |  |  |  |  |  |  |  |  |
| 2015 | 20,332 | 10 | — | — |  | 1,392,627 | 36,576 | — | — |  |
| 2016 | 159,450 | 129 | 1.9 | 1.00, 3.61 | 0.049 | 1,070,879 | 28,725 | 0.97 | 0.96, 0.99 | 0.002 |
| 2017 | 165,158 | 4,599 | 62.1 | 33.5, 115 | <0.001 | 166,600 | 10,068 | 1.56 | 1.52, 1.60 | <0.001 |
| 2018 | 73,581 | 1,895 | 73.6 | 39.6, 137 | <0.001 | 201,847 | 13,309 | 1.78 | 1.74, 1.82 | <0.001 |
| 2019 | 104,373 | 2,412 | 75.8 | 40.8, 141 | <0.001 | 1,310,289 | 50,871 | 1.41 | 1.39, 1.43 | <0.001 |
| 2020 | 42,677 | 998 | 55.7 | 30.0, 104 | <0.001 | 781,894 | 28,836 | 1.13 | 1.11, 1.15 | <0.001 |
| 2021 | 69,902 | 1,493 | 51.7 | 27.8, 96.0 | <0.001 | 447,322 | 15,359 | 0.96 | 0.94, 0.98 | <0.001 |
| 2022 | 147,441 | 2,876 | 43.2 | 23.3, 80.3 | <0.001 | 1,242,066 | 47,348 | 1.09 | 1.08, 1.11 | <0.001 |
| 2023 | 125,385 | 2,742 | 63.7 | 34.3, 118 | <0.001 | 1,300,384 | 46,636 | 1.24 | 1.22, 1.26 | <0.001 |
| 2024 | 216,910 | 4,182 | 60.7 | 32.7, 113 | <0.001 | 5,827,068 | 178,129 | 1.1 | 1.09, 1.11 | <0.001 |
| **Geographic region** | |  |  |  |  |  |  |  |  |  |
| North | 39,870 | 1,762 | — | — |  | 289,076 | 18,440 | — | — |  |
| Northeast | 565,219 | 10,473 | 0.57 | 0.54, 0.60 | <0.001 | 1,298,841 | 67,312 | 0.8 | 0.79, 0.81 | <0.001 |
| Southeast | 454,973 | 7,394 | 0.33 | 0.31, 0.35 | <0.001 | 7,947,511 | 187,728 | 0.33 | 0.33, 0.34 | <0.001 |
| South | 3,360 | 219 | 1.55 | 1.33, 1.80 | <0.001 | 2,237,124 | 75,762 | 0.47 | 0.46, 0.48 | <0.001 |
| Central west | 61,787 | 1,488 | 0.48 | 0.44, 0.51 | <0.001 | 1,968,424 | 106,615 | 0.81 | 0.79, 0.82 | <0.001 |
| **Sex** |  |  |  |  |  |  |  |  |  |  |
| Female | 691,264 | 12,106 | — | — |  | 7,539,897 | 245,655 | — | — |  |
| Male | 433,945 | 9,230 | 1.13 | 1.10, 1.16 | <0.001 | 6,201,079 | 210,202 | 1.06 | 1.06, 1.07 | <0.001 |
| **Age group** |  |  |  |  |  |  |  |  |  |  |
| 20-39 | 377,567 | 5,240 | — | — |  | 4,933,387 | 118,460 | — | — |  |
| <1 | 10,116 | 1,037 | 8.43 | 7.85, 9.06 | <0.001 | 126,351 | 9,393 | 2.93 | 2.86, 2.99 | <0.001 |
| 1-9 | 59,615 | 3,050 | 3.85 | 3.68, 4.03 | <0.001 | 1,040,643 | 46,951 | 1.84 | 1.82, 1.86 | <0.001 |
| 10-19 | 125,323 | 2,679 | 1.56 | 1.49, 1.63 | <0.001 | 2,154,882 | 63,447 | 1.23 | 1.21, 1.24 | <0.001 |
| 40-49 | 190,817 | 2,412 | 0.87 | 0.83, 0.91 | <0.001 | 2,059,198 | 55,934 | 1.12 | 1.11, 1.13 | <0.001 |
| 50-59 | 163,077 | 2,225 | 0.89 | 0.85, 0.94 | <0.001 | 1,627,483 | 51,433 | 1.26 | 1.25, 1.28 | <0.001 |
| 60-69 | 113,983 | 1,926 | 1.04 | 0.99, 1.10 | 0.14 | 1,071,528 | 45,922 | 1.67 | 1.65, 1.69 | <0.001 |
| 70-79 | 60,352 | 1,548 | 1.57 | 1.48, 1.67 | <0.001 | 527,676 | 37,534 | 2.74 | 2.71, 2.78 | <0.001 |
| 80-89 | 21,269 | 997 | 2.99 | 2.78, 3.21 | <0.001 | 174,881 | 21,982 | 5.13 | 5.05, 5.21 | <0.001 |
| ≥90 | 3,090 | 222 | 5.02 | 4.35, 5.79 | <0.001 | 24,947 | 4,801 | 8.7 | 8.42, 9.00 | <0.001 |
| **Diabetes** |  |  |  |  |  |  |  |  |  |  |
| No | 1,083,637 | 19,606 | — | — |  | 13,291,238 | 421,446 | — | — |  |
| Yes | 41,572 | 1,730 | 1.51 | 1.42, 1.60 | <0.001 | 449,738 | 34,411 | 1.45 | 1.43, 1.47 | <0.001 |
| **Autoimmune disease** | |  |  |  |  |  |  |  |  |  |
| No | 1,119,668 | 21,032 | — | — |  | 13,667,310 | 450,307 | — | — |  |
| Yes | 5,541 | 304 | 1.64 | 1.42, 1.89 | <0.001 | 73,666 | 5,550 | 1.4 | 1.35, 1.45 | <0.001 |
| **Hematologic disease** | |  |  |  |  |  |  |  |  |  |
| No | 1,120,854 | 21,109 | — | — |  | 13,677,675 | 450,851 | — | — |  |
| Yes | 4,355 | 227 | 0.94 | 0.78, 1.13 | 0.5 | 63,301 | 5,006 | 1.29 | 1.24, 1.33 | <0.001 |
| **Liver disease** | |  |  |  |  |  |  |  |  |  |
| No | 1,120,486 | 21,078 | — | — |  | 13,677,222 | 451,321 | — | — |  |
| Yes | 4,723 | 258 | 1.1 | 0.93, 1.31 | 0.3 | 63,754 | 4,536 | 0.98 | 0.94, 1.02 | 0.3 |
| **Hypertension** | |  |  |  |  |  |  |  |  |  |
| No | 1,022,690 | 17,773 | — | — |  | 12,680,560 | 385,151 | — | — |  |
| Yes | 102,519 | 3,563 | 1.65 | 1.57, 1.72 | <0.001 | 1,060,416 | 70,706 | 1.56 | 1.54, 1.57 | <0.001 |
| **Kidney disease** | |  |  |  |  |  |  |  |  |  |
| No | 1,120,902 | 20,983 | — | — |  | 13,682,901 | 449,432 | — | — |  |
| Yes | 4,307 | 353 | 2.18 | 1.90, 2.50 | <0.001 | 58,075 | 6,425 | 1.86 | 1.80, 1.92 | <0.001 |

Abbreviations: CI = Confidence Interval, OR = Odds Ratio

# Supplementary Table 2: Risk factors for death after chikungunya estimated using Fine and Gray model. N refers to the number of cases in each subgroup; sHR = sub-distribution hazard ratio

|  | **Chikungunya** | | | | | **Other causes** | | | | |
| --- | --- | --- | --- | --- | --- | --- | --- | --- | --- | --- |
| **Characteristic** | **N** | **Event N** | **sHR** | **95% CI** | **p-value** | **N** | **Event N** | **sHR** | **95% CI** | **p-value** |
| **Year** |  |  |  |  | <0.001 |  |  |  |  | <0.001 |
| 2015 | 10 | 1 | — | — |  | 10 | 0 | — | — |  |
| 2016 | 129 | 24 | 1.1 | 0.13, 9.36 |  | 129 | 1 | 98.2 | 11.0, 881 |  |
| 2017 | 4,599 | 176 | 0.32 | 0.04, 2.58 |  | 4,599 | 42 | 147 | 50.8, 425 |  |
| 2018 | 1,895 | 42 | 0.19 | 0.02, 1.53 |  | 1,895 | 18 | 167 | 54.1, 514 |  |
| 2019 | 2,412 | 88 | 0.24 | 0.03, 1.95 |  | 2,412 | 22 | 155 | 51.6, 467 |  |
| 2020 | 998 | 22 | 0.14 | 0.02, 1.17 |  | 998 | 27 | 458 | 154, 1,359 |  |
| 2021 | 1,493 | 13 | 0.06 | 0.01, 0.56 |  | 1,493 | 23 | 264 | 88.5, 785 |  |
| 2022 | 2,876 | 71 | 0.16 | 0.02, 1.33 |  | 2,876 | 26 | 142 | 48.4, 417 |  |
| 2023 | 2,742 | 107 | 0.23 | 0.03, 1.89 |  | 2,742 | 64 | 364 | 129, 1,032 |  |
| 2024 | 4,182 | 184 | 0.21 | 0.03, 1.75 |  | 4,182 | 93 | 320 | 110, 932 |  |
| **Geographic region** | |  |  |  | <0.001 |  |  |  |  | 0.11 |
| North | 1,762 | 9 | — | — |  | 1,762 | 24 | — | — |  |
| Northeast | 10,473 | 343 | 5.81 | 2.98, 11.3 |  | 10,473 | 133 | 0.7 | 0.44, 1.12 |  |
| Southeast | 7,394 | 313 | 6.2 | 3.16, 12.2 |  | 7,394 | 117 | 0.63 | 0.38, 1.04 |  |
| South | 219 | 5 | 3.22 | 1.08, 9.64 |  | 219 | 5 | 0.76 | 0.29, 1.99 |  |
| Central west | 1,488 | 58 | 6.56 | 3.18, 13.5 |  | 1,488 | 37 | 0.98 | 0.55, 1.77 |  |
| **Sex** |  |  |  |  | <0.001 |  |  |  |  | 0.025 |
| Female | 12,106 | 341 | — | — |  | 12,106 | 161 | — | — |  |
| Male | 9,230 | 387 | 1.52 | 1.31, 1.77 |  | 9,230 | 155 | 1.3 | 1.03, 1.63 |  |
| **Age group** |  |  |  |  | <0.001 |  |  |  |  | <0.001 |
| 20-39 | 5,240 | 85 | — | — |  | 5,240 | 55 | — | — |  |
| <1 | 1,037 | 38 | 2.19 | 1.48, 3.25 |  | 1,037 | 19 | 1.68 | 0.98, 2.86 |  |
| 1-9 | 3,050 | 20 | 0.4 | 0.25, 0.65 |  | 3,050 | 26 | 0.76 | 0.47, 1.22 |  |
| 10-19 | 2,679 | 35 | 0.78 | 0.52, 1.16 |  | 2,679 | 25 | 0.89 | 0.55, 1.43 |  |
| 40-49 | 2,412 | 52 | 1.26 | 0.89, 1.78 |  | 2,412 | 33 | 1.25 | 0.81, 1.93 |  |
| 50-59 | 2,225 | 63 | 1.47 | 1.05, 2.05 |  | 2,225 | 39 | 1.51 | 1.0, 2.28 |  |
| 60-69 | 1,926 | 83 | 1.96 | 1.43, 2.69 |  | 1,926 | 43 | 1.68 | 1.10, 2.55 |  |
| 70-79 | 1,548 | 145 | 4.01 | 3.00, 5.36 |  | 1,548 | 26 | 1.18 | 0.72, 1.94 |  |
| 80-89 | 997 | 158 | 6.97 | 5.24, 9.28 |  | 997 | 37 | 2.66 | 1.66, 4.26 |  |
| ≥90 | 222 | 49 | 10 | 6.82, 14.7 |  | 222 | 13 | 4.4 | 2.35, 8.25 |  |
| **Diabetes** |  |  |  |  | <0.001 |  |  |  |  | 0.072 |
| No | 19,606 | 537 | — | — |  | 19,606 | 260 | — | — |  |
| Yes | 1,730 | 191 | 1.78 | 1.47, 2.17 |  | 1,730 | 56 | 1.38 | 0.97, 1.97 |  |
| **Autoimmune disease** | |  |  |  | 0.026 |  |  |  |  | 0.024 |
| No | 21,032 | 708 | — | — |  | 21,032 | 302 | — | — |  |
| Yes | 304 | 20 | 1.75 | 1.07, 2.88 |  | 304 | 14 | 2.21 | 1.11, 4.41 |  |
| **Hematologic disease** | |  |  |  | 0.04 |  |  |  |  | 0.2 |
| No | 21,109 | 720 | — | — |  | 21,109 | 306 | — | — |  |
| Yes | 227 | 8 | 0.38 | 0.15, 0.96 |  | 227 | 10 | 1.78 | 0.76, 4.21 |  |
| **Liver disease** | |  |  |  | <0.001 |  |  |  |  | 0.4 |
| No | 21,078 | 702 | — | — |  | 21,078 | 305 | — | — |  |
| Yes | 258 | 26 | 2.58 | 1.61, 4.13 |  | 258 | 11 | 1.47 | 0.65, 3.34 |  |
| **Hypertension** | |  |  |  | 0.06 |  |  |  |  | 0.14 |
| No | 17,773 | 424 | — | — |  | 17,773 | 218 | — | — |  |
| Yes | 3,563 | 304 | 1.2 | 0.99, 1.44 |  | 3,563 | 98 | 1.27 | 0.93, 1.75 |  |
| **Kidney disease** | |  |  |  | <0.001 |  |  |  |  | 0.2 |
| No | 20,983 | 671 | — | — |  | 20,983 | 300 | — | — |  |
| Yes | 353 | 57 | 1.87 | 1.39, 2.52 |  | 353 | 16 | 1.47 | 0.79, 2.74 |  |

# Supplementary Table 3 – Years of life lost (YLL) and average years of life lost (aYLL) for chikungunya inpatient deaths (all-cause) stratified by sex, geographic region and race/ethnicity.

| **Group** | **Mean age at death** | **Number of deaths** | **YLL** | **aYLL** |
| --- | --- | --- | --- | --- |
| Overall | 55.3 | 1,044 | 16743.6 | 16.0 |
| Male | 58.1 | 542 | 7849.9 | 14.5 |
| Female | 52.4 | 502 | 8758.1 | 17.4 |
| North | 36.5 | 33 | 728.7 | 22.1 |
| Northeast | 51.7 | 476 | 8238.2 | 17.3 |
| Southeast | 61.2 | 430 | 6077.4 | 14.1 |
| South | 72.2 | 10 | 103.4 | 10.3 |
| Central west | 51.5 | 95 | 1635.8 | 17.2 |
| Asian | 47.3 | 7 | 135.9 | 19.4 |
| White | 63.9 | 247 | 3223.1 | 13.0 |
| Indigenous | 35.8 | 4 | 92.3 | 23.1 |
| Mixed | 51.8 | 629 | 10915.5 | 17.4 |
| Black | 39.5 | 45 | 991.3 | 22.0 |

# Supplementary Table 4: Risk factors for death after dengue estimated using Fine and Gray model. sHR = sub-distribution hazard ratio

|  | **Dengue** | | | | | **Other Causes** | | | | |
| --- | --- | --- | --- | --- | --- | --- | --- | --- | --- | --- |
| **Characteristic** | **N** | **Event N** | **sHR** | **95% CI** | **p-value** | **N** | **Event N** | **sHR** | **95% CI** | **p-value** |
| **Year** |  |  |  |  | <0.001 |  |  |  |  | <0.001 |
| 2015 | 36,576 | 747 | — | — |  | 36,576 | 181 | — | — |  |
| 2016 | 28,725 | 601 | 0.97 | 0.88, 1.08 |  | 28,725 | 207 | 1.38 | 1.13, 1.69 |  |
| 2017 | 10,068 | 148 | 0.77 | 0.65, 0.92 |  | 10,068 | 89 | 1.89 | 1.46, 2.44 |  |
| 2018 | 13,309 | 174 | 0.64 | 0.55, 0.76 |  | 13,309 | 65 | 1.04 | 0.78, 1.39 |  |
| 2019 | 50,871 | 707 | 0.55 | 0.49, 0.61 |  | 50,871 | 203 | 0.68 | 0.56, 0.84 |  |
| 2020 | 28,836 | 478 | 0.6 | 0.54, 0.68 |  | 28,836 | 222 | 1.36 | 1.11, 1.66 |  |
| 2021 | 15,359 | 225 | 0.67 | 0.58, 0.78 |  | 15,359 | 140 | 1.77 | 1.42, 2.22 |  |
| 2022 | 47,348 | 882 | 0.7 | 0.63, 0.77 |  | 47,348 | 338 | 1.24 | 1.03, 1.49 |  |
| 2023 | 46,636 | 936 | 0.66 | 0.60, 0.73 |  | 46,636 | 305 | 1.03 | 0.85, 1.24 |  |
| 2024 | 178,129 | 5,087 | 0.81 | 0.75, 0.88 |  | 178,129 | 1,230 | 0.93 | 0.79, 1.09 |  |
| **Geographic region** | |  |  |  | <0.001 |  |  |  |  | <0.001 |
| North | 18,440 | 182 | — | — |  | 18,440 | 118 | — | — |  |
| Northeast | 67,312 | 842 | 1.23 | 1.04, 1.44 |  | 67,312 | 391 | 0.98 | 0.79, 1.20 |  |
| Southeast | 187,728 | 5,280 | 1.61 | 1.39, 1.87 |  | 187,728 | 1,558 | 0.92 | 0.75, 1.11 |  |
| South | 75,762 | 1,897 | 1.35 | 1.16, 1.58 |  | 75,762 | 427 | 0.55 | 0.45, 0.68 |  |
| Central west | 106,615 | 1,784 | 1.22 | 1.05, 1.43 |  | 106,615 | 486 | 0.56 | 0.46, 0.69 |  |
| **Sex** |  |  |  |  | <0.001 |  |  |  |  | <0.001 |
| Female | 245,655 | 4,954 | — | — |  | 245,655 | 1,319 | — | — |  |
| Male | 210,202 | 5,031 | 1.24 | 1.20, 1.29 |  | 210,202 | 1,661 | 1.52 | 1.41, 1.63 |  |
| **Age group** |  |  |  |  | <0.001 |  |  |  |  | <0.001 |
| 20-39 | 118,460 | 1,263 | — | — |  | 118,460 | 368 | — | — |  |
| <1 | 9,393 | 109 | 1.11 | 0.91, 1.35 |  | 9,393 | 48 | 1.51 | 1.11, 2.04 |  |
| 1-9 | 46,951 | 223 | 0.46 | 0.40, 0.53 |  | 46,951 | 81 | 0.51 | 0.40, 0.65 |  |
| 10-19 | 63,447 | 368 | 0.55 | 0.49, 0.61 |  | 63,447 | 98 | 0.46 | 0.37, 0.58 |  |
| 40-49 | 55,934 | 900 | 1.4 | 1.29, 1.53 |  | 55,934 | 261 | 1.46 | 1.25, 1.71 |  |
| 50-59 | 51,433 | 1,144 | 1.74 | 1.61, 1.89 |  | 51,433 | 362 | 2.06 | 1.78, 2.38 |  |
| 60-69 | 45,922 | 1,488 | 2.27 | 2.10, 2.45 |  | 45,922 | 488 | 2.93 | 2.55, 3.37 |  |
| 70-79 | 37,534 | 1,955 | 3.34 | 3.10, 3.60 |  | 37,534 | 532 | 3.73 | 3.24, 4.29 |  |
| 80-89 | 21,982 | 1,862 | 5.34 | 4.95, 5.77 |  | 21,982 | 558 | 6.71 | 5.83, 7.73 |  |
| ≥90 | 4,801 | 673 | 9.31 | 8.45, 10.3 |  | 4,801 | 184 | 10.5 | 8.71, 12.6 |  |
| **Diabetes** |  |  |  |  | <0.001 |  |  |  |  | <0.001 |
| No | 421,446 | 7,728 | — | — |  | 421,446 | 2,361 | — | — |  |
| Yes | 34,411 | 2,257 | 1.38 | 1.30, 1.46 |  | 34,411 | 619 | 1.29 | 1.16, 1.43 |  |
| **Autoimmune disease** | |  |  |  | <0.001 |  |  |  |  | <0.001 |
| No | 450,307 | 9,677 | — | — |  | 450,307 | 2,875 | — | — |  |
| Yes | 5,550 | 308 | 1.37 | 1.19, 1.59 |  | 5,550 | 105 | 1.57 | 1.22, 2.02 |  |
| **Hematologic disease** | |  |  |  | <0.001 |  |  |  |  | 0.016 |
| No | 450,851 | 9,631 | — | — |  | 450,851 | 2,885 | — | — |  |
| Yes | 5,006 | 354 | 1.97 | 1.71, 2.26 |  | 5,006 | 95 | 1.42 | 1.07, 1.90 |  |
| **Liver disease** | |  |  |  | 0.11 |  |  |  |  | <0.001 |
| No | 451,321 | 9,728 | — | — |  | 451,321 | 2,864 | — | — |  |
| Yes | 4,536 | 257 | 1.15 | 0.97, 1.36 |  | 4,536 | 116 | 1.96 | 1.52, 2.54 |  |
| **Hypertension** | |  |  |  | <0.001 |  |  |  |  | <0.001 |
| No | 385,151 | 5,795 | — | — |  | 385,151 | 1,842 | — | — |  |
| Yes | 70,706 | 4,190 | 1.59 | 1.52, 1.68 |  | 70,706 | 1,138 | 1.33 | 1.21, 1.46 |  |
| **Kidney disease** | |  |  |  | <0.001 |  |  |  |  | <0.001 |
| No | 449,432 | 9,283 | — | — |  | 449,432 | 2,760 | — | — |  |
| Yes | 6,425 | 702 | 2.14 | 1.95, 2.34 |  | 6,425 | 220 | 2.2 | 1.86, 2.60 |  |

# Supplementary Table 5 – Years of life lost (YLL) and average years of life lost (aYLL) for dengue inpatient deaths (all-cause) stratified by sex, geographic region and race/ethnicity.

| **Group** | **Mean age at death** | **Number of deaths** | **YLL** | **aYLL** |
| --- | --- | --- | --- | --- |
| Overall | 60.9 | 12,969 | 187614.7 | 14.5 |
| Male | 61.7 | 6,694 | 90327.5 | 13.5 |
| Female | 60 | 6,275 | 96471.5 | 15.4 |
| North | 46.9 | 300 | 5762.0 | 19.2 |
| Northeast | 44.3 | 1,233 | 24407.1 | 19.8 |
| Southeast | 63.4 | 6,838 | 93382.4 | 13.7 |
| South | 67.3 | 2,324 | 28014.8 | 12.1 |
| Central west | 57.6 | 2,270 | 35623.9 | 15.7 |
| Asian | 68.6 | 128 | 1640.8 | 12.8 |
| White | 65.9 | 6,435 | 81003.5 | 12.6 |
| Indigenous | 40.8 | 44 | 991.5 | 22.5 |
| Mixed | 53.5 | 4,185 | 72431.4 | 17.3 |
| Black | 56.8 | 600 | 10284.4 | 17.1 |

# Supplementary Table 6: Risk factors for hospitalisation for chikungunya and dengue using only laboratory-confirmed cases.

|  | **Chikungunya** | | | | | **Dengue** | | | | |
| --- | --- | --- | --- | --- | --- | --- | --- | --- | --- | --- |
| **Characteristic** | **N** | **Event N** | **OR** | **95% CI** | **p-value** | **N** | **Event N** | **OR** | **95% CI** | **p-value** |
| **Year** |  |  |  |  |  |  |  |  |  |  |
| 2015 | 4,061 | 3 | — | — |  | 535,096 | 21,770 | — | — |  |
| 2016 | 29,917 | 68 | 3.39 | 1.07, 10.8 | 0.039 | 306,065 | 13,056 | 1.03 | 1.00, 1.05 | 0.024 |
| 2017 | 49,575 | 2,198 | 60.3 | 19.4, 187 | <0.001 | 42,625 | 4,503 | 1.78 | 1.72, 1.84 | <0.001 |
| 2018 | 22,194 | 1,040 | 69.4 | 22.3, 216 | <0.001 | 62,985 | 6,836 | 1.93 | 1.88, 1.99 | <0.001 |
| 2019 | 31,596 | 1,251 | 68 | 21.9, 211 | <0.001 | 420,588 | 27,165 | 1.48 | 1.46, 1.51 | <0.001 |
| 2020 | 19,763 | 595 | 41 | 13.2, 128 | <0.001 | 314,548 | 17,074 | 1.03 | 1.01, 1.06 | 0.002 |
| 2021 | 31,266 | 1,020 | 46.2 | 14.9, 143 | <0.001 | 248,170 | 10,682 | 0.79 | 0.77, 0.81 | <0.001 |
| 2022 | 61,526 | 1,653 | 34.2 | 11.0, 106 | <0.001 | 579,275 | 30,476 | 0.94 | 0.93, 0.96 | <0.001 |
| 2023 | 53,896 | 1,846 | 51.3 | 16.5, 159 | <0.001 | 618,788 | 32,320 | 1.11 | 1.09, 1.13 | <0.001 |
| 2024 | 95,329 | 2,569 | 41 | 13.2, 127 | <0.001 | 2,146,800 | 103,066 | 1.08 | 1.07, 1.10 | <0.001 |
| **Geographic region** | |  |  |  |  |  |  |  |  |  |
| North | 23,215 | 1,129 | — | — |  | 137,334 | 11,412 | — | — |  |
| Northeast | 169,436 | 5,364 | 0.82 | 0.77, 0.88 | <0.001 | 312,821 | 29,347 | 1.09 | 1.06, 1.11 | <0.001 |
| Southeast | 172,675 | 4,397 | 0.57 | 0.53, 0.62 | <0.001 | 3,143,264 | 114,933 | 0.38 | 0.37, 0.39 | <0.001 |
| South | 2,617 | 204 | 2.07 | 1.76, 2.44 | <0.001 | 930,736 | 50,144 | 0.58 | 0.57, 0.59 | <0.001 |
| Central west | 31,180 | 1,149 | 0.9 | 0.82, 0.99 | 0.027 | 750,785 | 61,112 | 0.93 | 0.91, 0.95 | <0.001 |
| **Sex** |  |  |  |  |  |  |  |  |  |  |
| Female | 259,463 | 6,901 | — | — |  | 2,881,210 | 142,960 | — | — |  |
| Male | 139,660 | 5,342 | 1.29 | 1.24, 1.34 | <0.001 | 2,393,730 | 123,988 | 1.07 | 1.06, 1.07 | <0.001 |
| **Age group** |  |  |  |  |  |  |  |  |  |  |
| 20-39 | 113,681 | 2,765 | — | — |  | 1,686,121 | 65,189 | — | — |  |
| <1 | 3,941 | 635 | 7.76 | 7.05, 8.53 | <0.001 | 45,432 | 5,327 | 2.89 | 2.80, 2.98 | <0.001 |
| 1-9 | 20,835 | 1,850 | 3.84 | 3.61, 4.08 | <0.001 | 396,773 | 26,779 | 1.7 | 1.67, 1.72 | <0.001 |
| 10-19 | 37,151 | 1,479 | 1.63 | 1.53, 1.74 | <0.001 | 760,354 | 35,983 | 1.22 | 1.20, 1.24 | <0.001 |
| 40-49 | 70,588 | 1,353 | 0.75 | 0.70, 0.80 | <0.001 | 830,318 | 32,719 | 1 | 0.98, 1.01 | 0.8 |
| 50-59 | 65,629 | 1,269 | 0.71 | 0.66, 0.76 | <0.001 | 701,403 | 30,918 | 1.08 | 1.06, 1.09 | <0.001 |
| 60-69 | 50,029 | 1,153 | 0.78 | 0.73, 0.84 | <0.001 | 496,630 | 28,418 | 1.36 | 1.34, 1.38 | <0.001 |
| 70-79 | 26,736 | 968 | 1.2 | 1.11, 1.30 | <0.001 | 256,987 | 24,115 | 2.22 | 2.19, 2.26 | <0.001 |
| 80-89 | 9,167 | 631 | 2.38 | 2.17, 2.61 | <0.001 | 87,968 | 14,364 | 4.18 | 4.09, 4.26 | <0.001 |
| ≥90 | 1,366 | 140 | 3.86 | 3.21, 4.64 | <0.001 | 12,954 | 3,136 | 6.98 | 6.69, 7.28 | <0.001 |
| **Diabetes** |  |  |  |  |  |  |  |  |  |  |
| No | 380,037 | 11,103 | — | — |  | 5,072,496 | 244,949 | — | — |  |
| Yes | 19,086 | 1,140 | 1.58 | 1.47, 1.70 | <0.001 | 202,444 | 21,999 | 1.46 | 1.44, 1.49 | <0.001 |
| **Autoimmune disease** | |  |  |  |  |  |  |  |  |  |
| No | 396,364 | 12,028 | — | — |  | 5,243,934 | 263,358 | — | — |  |
| Yes | 2,759 | 215 | 1.63 | 1.37, 1.93 | <0.001 | 31,006 | 3,590 | 1.48 | 1.42, 1.54 | <0.001 |
| **Hematologic disease** | |  |  |  |  |  |  |  |  |  |
| No | 397,180 | 12,083 | — | — |  | 5,248,774 | 263,792 | — | — |  |
| Yes | 1,943 | 160 | 1.09 | 0.87, 1.36 | 0.5 | 26,166 | 3,156 | 1.3 | 1.24, 1.36 | <0.001 |
| **Liver disease** | |  |  |  |  |  |  |  |  |  |
| No | 396,928 | 12,064 | — | — |  | 5,249,129 | 264,110 | — | — |  |
| Yes | 2,195 | 179 | 1.14 | 0.93, 1.40 | 0.2 | 25,811 | 2,838 | 1.03 | 0.98, 1.08 | 0.3 |
| **Hypertension** | |  |  |  |  |  |  |  |  |  |
| No | 353,100 | 9,984 | — | — |  | 4,796,563 | 221,524 | — | — |  |
| Yes | 46,023 | 2,259 | 1.71 | 1.61, 1.81 | <0.001 | 478,377 | 45,424 | 1.58 | 1.56, 1.61 | <0.001 |
| **Kidney disease** | |  |  |  |  |  |  |  |  |  |
| No | 397,077 | 11,989 | — | — |  | 5,250,002 | 262,706 | — | — |  |
| Yes | 2,046 | 254 | 2.23 | 1.89, 2.63 | <0.001 | 24,938 | 4,242 | 1.98 | 1.90, 2.06 | <0.001 |

# Supplementary Table 7: Risk factors for death following chikungunya estimated using Fine and Gray model, using only laboratory-confirmed cases.

|  | **Chikungunya** | | | | | **Other causes** | | | | |
| --- | --- | --- | --- | --- | --- | --- | --- | --- | --- | --- |
| **Characteristic** | **N** | **Event N** | **sHR** | **95% CI** | **p-value** | **N** | **Event N** | **sHR** | **95% CI** | **p-value** |
| **Year** |  |  |  |  | <0.001 |  |  |  |  | <0.001 |
| 2015 | 3 | 0 | — | — |  | 3 | 0 | — | — |  |
| 2016 | 68 | 14 | 395 | 102, 1,532 |  | 68 | 1 | 497 | 47.9, 5,149 |  |
| 2017 | 2,198 | 151 | 190 | 55.7, 645 |  | 2,198 | 25 | 455 | 123, 1,688 |  |
| 2018 | 1,040 | 39 | 121 | 34.3, 426 |  | 1,040 | 13 | 511 | 129, 2,022 |  |
| 2019 | 1,251 | 73 | 130 | 37.6, 449 |  | 1,251 | 14 | 437 | 113, 1,684 |  |
| 2020 | 595 | 21 | 71.9 | 19.7, 263 |  | 595 | 23 | 1,601 | 432, 5,930 |  |
| 2021 | 1,020 | 13 | 33.9 | 8.99, 128 |  | 1,020 | 22 | 914 | 247, 3,384 |  |
| 2022 | 1,653 | 65 | 92.4 | 26.7, 320 |  | 1,653 | 22 | 531 | 142, 1,982 |  |
| 2023 | 1,846 | 100 | 127 | 36.8, 435 |  | 1,846 | 45 | 934 | 260, 3,351 |  |
| 2024 | 2,569 | 178 | 142 | 41.3, 486 |  | 2,569 | 74 | 1,042 | 289, 3,754 |  |
| **Geographic region** | |  |  |  | <0.001 |  |  |  |  | 0.7 |
| North | 1,129 | 8 | — | — |  | 1,129 | 20 | — | — |  |
| Northeast | 5,364 | 295 | 6.85 | 3.38, 13.9 |  | 5,364 | 90 | 0.72 | 0.43, 1.20 |  |
| Southeast | 4,397 | 291 | 6.48 | 3.19, 13.2 |  | 4,397 | 95 | 0.75 | 0.44, 1.28 |  |
| South | 204 | 5 | 2.47 | 0.81, 7.54 |  | 204 | 5 | 0.78 | 0.29, 2.08 |  |
| Central west | 1,149 | 55 | 5.39 | 2.53, 11.5 |  | 1,149 | 29 | 0.86 | 0.46, 1.58 |  |
| **Sex** |  |  |  |  | <0.001 |  |  |  |  | 0.025 |
| Female | 6,901 | 307 | — | — |  | 6,901 | 117 | — | — |  |
| Male | 5,342 | 347 | 1.46 | 1.25, 1.71 |  | 5,342 | 122 | 1.35 | 1.04, 1.76 |  |
| **Age group** |  |  |  |  | <0.001 |  |  |  |  | 0.022 |
| 20-39 | 2,765 | 81 | — | — |  | 2,765 | 46 | — | — |  |
| <1 | 635 | 33 | 1.61 | 1.06, 2.44 |  | 635 | 13 | 1.16 | 0.62, 2.17 |  |
| 1-9 | 1,850 | 19 | 0.33 | 0.20, 0.55 |  | 1,850 | 21 | 0.63 | 0.37, 1.07 |  |
| 10-19 | 1,479 | 34 | 0.72 | 0.48, 1.09 |  | 1,479 | 21 | 0.84 | 0.50, 1.41 |  |
| 40-49 | 1,353 | 48 | 1.12 | 0.78, 1.61 |  | 1,353 | 20 | 0.84 | 0.50, 1.41 |  |
| 50-59 | 1,269 | 59 | 1.29 | 0.92, 1.82 |  | 1,269 | 29 | 1.22 | 0.77, 1.95 |  |
| 60-69 | 1,153 | 75 | 1.63 | 1.18, 2.26 |  | 1,153 | 37 | 1.5 | 0.95, 2.38 |  |
| 70-79 | 968 | 126 | 3.06 | 2.27, 4.13 |  | 968 | 20 | 0.9 | 0.51, 1.59 |  |
| 80-89 | 631 | 140 | 5.36 | 3.99, 7.20 |  | 631 | 25 | 1.77 | 1.02, 3.07 |  |
| ≥90 | 140 | 39 | 6.9 | 4.54, 10.5 |  | 140 | 7 | 2.24 | 0.98, 5.13 |  |
| **Diabetes** |  |  |  |  | <0.001 |  |  |  |  | 0.3 |
| No | 11,103 | 479 | — | — |  | 11,103 | 196 | — | — |  |
| Yes | 1,140 | 175 | 1.74 | 1.43, 2.13 |  | 1,140 | 43 | 1.26 | 0.84, 1.89 |  |
| **Autoimmune disease** | |  |  |  | 0.053 |  |  |  |  | 0.1 |
| No | 12,028 | 635 | — | — |  | 12,028 | 228 | — | — |  |
| Yes | 215 | 19 | 1.63 | 0.99, 2.66 |  | 215 | 11 | 1.88 | 0.88, 4.02 |  |
| **Hematologic disease** | |  |  |  | 0.015 |  |  |  |  | 0.14 |
| No | 12,083 | 648 | — | — |  | 12,083 | 230 | — | — |  |
| Yes | 160 | 6 | 0.29 | 0.11, 0.78 |  | 160 | 9 | 1.94 | 0.80, 4.72 |  |
| **Liver disease** | |  |  |  | <0.001 |  |  |  |  | 0.5 |
| No | 12,064 | 629 | — | — |  | 12,064 | 230 | — | — |  |
| Yes | 179 | 25 | 2.67 | 1.69, 4.20 |  | 179 | 9 | 1.42 | 0.57, 3.54 |  |
| **Hypertension** | |  |  |  | 0.3 |  |  |  |  | 0.15 |
| No | 9,984 | 387 | — | — |  | 9,984 | 164 | — | — |  |
| Yes | 2,259 | 267 | 1.12 | 0.92, 1.35 |  | 2,259 | 75 | 1.31 | 0.91, 1.90 |  |
| **Kidney disease** | |  |  |  | <0.001 |  |  |  |  | 0.2 |
| No | 11,989 | 603 | — | — |  | 11,989 | 225 | — | — |  |
| Yes | 254 | 51 | 1.67 | 1.24, 2.26 |  | 254 | 14 | 1.5 | 0.78, 2.89 |  |

# Supplementary Table 8: Risk factors for death following dengue estimated using Fine and Gray model, using only laboratory-confirmed cases.

|  | **Dengue** | | | | | **Other causes** | | | | |
| --- | --- | --- | --- | --- | --- | --- | --- | --- | --- | --- |
| **Characteristic** | **N** | **Event N** | **sHR** | **95% CI** | **p-value** | **N** | **Event N** | **sHR** | **95% CI** | **p-value** |
| **Year** |  |  |  |  | <0.001 |  |  |  |  | <0.001 |
| 2015 | 21,770 | 703 | — | — |  | 21,770 | 138 | — | — |  |
| 2016 | 13,056 | 521 | 1.11 | 0.99, 1.24 |  | 13,056 | 119 | 1.3 | 1.02, 1.67 |  |
| 2017 | 4,503 | 125 | 0.89 | 0.74, 1.08 |  | 4,503 | 54 | 1.91 | 1.38, 2.63 |  |
| 2018 | 6,836 | 143 | 0.66 | 0.55, 0.79 |  | 6,836 | 35 | 0.83 | 0.57, 1.21 |  |
| 2019 | 27,165 | 623 | 0.58 | 0.52, 0.64 |  | 27,165 | 118 | 0.59 | 0.46, 0.75 |  |
| 2020 | 17,074 | 403 | 0.59 | 0.52, 0.67 |  | 17,074 | 169 | 1.37 | 1.09, 1.72 |  |
| 2021 | 10,682 | 187 | 0.52 | 0.44, 0.61 |  | 10,682 | 118 | 1.71 | 1.33, 2.20 |  |
| 2022 | 30,476 | 767 | 0.63 | 0.57, 0.70 |  | 30,476 | 274 | 1.22 | 0.99, 1.50 |  |
| 2023 | 32,320 | 785 | 0.56 | 0.50, 0.62 |  | 32,320 | 237 | 0.94 | 0.76, 1.17 |  |
| 2024 | 103,066 | 3,805 | 0.72 | 0.66, 0.78 |  | 103,066 | 746 | 0.79 | 0.66, 0.96 |  |
| **Geographic region** | |  |  |  | <0.001 |  |  |  |  | <0.001 |
| North | 11,412 | 158 | — | — |  | 11,412 | 91 | — | — |  |
| Northeast | 29,347 | 667 | 1.63 | 1.37, 1.94 |  | 29,347 | 195 | 0.91 | 0.71, 1.17 |  |
| Southeast | 114,933 | 4,385 | 1.61 | 1.37, 1.89 |  | 114,933 | 1,026 | 0.79 | 0.63, 0.98 |  |
| South | 50,144 | 1,624 | 1.33 | 1.13, 1.57 |  | 50,144 | 338 | 0.55 | 0.43, 0.70 |  |
| Central west | 61,112 | 1,228 | 1.06 | 0.90, 1.25 |  | 61,112 | 358 | 0.56 | 0.44, 0.71 |  |
| **Sex** |  |  |  |  | <0.001 |  |  |  |  | <0.001 |
| Female | 142,960 | 3,974 | — | — |  | 142,960 | 872 | — | — |  |
| Male | 123,988 | 4,088 | 1.25 | 1.19, 1.30 |  | 123,988 | 1,136 | 1.55 | 1.42, 1.69 |  |
| **Age group** |  |  |  |  | <0.001 |  |  |  |  | <0.001 |
| 20-39 | 65,189 | 1,035 | — | — |  | 65,189 | 236 | — | — |  |
| <1 | 5,327 | 91 | 1.09 | 0.88, 1.35 |  | 5,327 | 23 | 1.09 | 0.71, 1.67 |  |
| 1-9 | 26,779 | 192 | 0.46 | 0.40, 0.54 |  | 26,779 | 41 | 0.39 | 0.28, 0.54 |  |
| 10-19 | 35,983 | 309 | 0.54 | 0.47, 0.61 |  | 35,983 | 61 | 0.43 | 0.32, 0.57 |  |
| 40-49 | 32,719 | 715 | 1.3 | 1.18, 1.43 |  | 32,719 | 177 | 1.45 | 1.19, 1.76 |  |
| 50-59 | 30,918 | 929 | 1.61 | 1.47, 1.76 |  | 30,918 | 248 | 2.01 | 1.68, 2.40 |  |
| 60-69 | 28,418 | 1,199 | 2.05 | 1.88, 2.23 |  | 28,418 | 321 | 2.72 | 2.29, 3.23 |  |
| 70-79 | 24,115 | 1,584 | 2.96 | 2.72, 3.21 |  | 24,115 | 380 | 3.66 | 3.08, 4.34 |  |
| 80-89 | 14,364 | 1,492 | 4.62 | 4.24, 5.03 |  | 14,364 | 394 | 6.44 | 5.42, 7.64 |  |
| ≥90 | 3,136 | 516 | 7.8 | 6.99, 8.71 |  | 3,136 | 127 | 10 | 8.03, 12.5 |  |
| **Diabetes** |  |  |  |  | <0.001 |  |  |  |  | <0.001 |
| No | 244,949 | 6,278 | — | — |  | 244,949 | 1,578 | — | — |  |
| Yes | 21,999 | 1,784 | 1.37 | 1.28, 1.45 |  | 21,999 | 430 | 1.31 | 1.15, 1.49 |  |
| **Autoimmune disease** | |  |  |  | 0.001 |  |  |  |  | <0.001 |
| No | 263,358 | 7,825 | — | — |  | 263,358 | 1,929 | — | — |  |
| Yes | 3,590 | 237 | 1.29 | 1.10, 1.52 |  | 3,590 | 79 | 1.77 | 1.34, 2.34 |  |
| **Hematologic disease** | |  |  |  | <0.001 |  |  |  |  | 0.055 |
| No | 263,792 | 7,786 | — | — |  | 263,792 | 1,944 | — | — |  |
| Yes | 3,156 | 276 | 1.97 | 1.69, 2.29 |  | 3,156 | 64 | 1.4 | 0.99, 1.96 |  |
| **Liver disease** | |  |  |  | 0.064 |  |  |  |  | <0.001 |
| No | 264,110 | 7,857 | — | — |  | 264,110 | 1,927 | — | — |  |
| Yes | 2,838 | 205 | 1.19 | 0.99, 1.42 |  | 2,838 | 81 | 2 | 1.49, 2.70 |  |
| **Hypertension** | |  |  |  | <0.001 |  |  |  |  | <0.001 |
| No | 221,524 | 4,731 | — | — |  | 221,524 | 1,230 | — | — |  |
| Yes | 45,424 | 3,331 | 1.57 | 1.48, 1.66 |  | 45,424 | 778 | 1.25 | 1.12, 1.40 |  |
| **Kidney disease** | |  |  |  | <0.001 |  |  |  |  | <0.001 |
| No | 262,706 | 7,499 | — | — |  | 262,706 | 1,856 | — | — |  |
| Yes | 4,242 | 563 | 2.07 | 1.88, 2.28 |  | 4,242 | 152 | 2.08 | 1.70, 2.54 |  |

# Supplementary Table 9: Risk factors for death following chikungunya estimated using Fine and Gray model stratified by period of COVID-19 pandemic. Pre-COVID (2015-2019), During COVID-19 (2020-2022) and Post COVID-19 (2023-2024). sHR = sub-distribution hazard ratio; CI = Confidence Interval

**Death: Chikungunya**

|  | **Pre** | | | | | **During** | | | | | **Post** | | | | |
| --- | --- | --- | --- | --- | --- | --- | --- | --- | --- | --- | --- | --- | --- | --- | --- |
| **Characteristic** | **N** | **Event N** | **sHR** | **95% CI** | **p-value** | **N** | **Event N** | **sHR** | **95% CI** | **p-value** | **N** | **Event N** | **sHR** | **95% CI** | **p-value** |
| **Year** |  |  |  |  | <0.001 |  |  |  |  |  |  |  |  |  |  |
| 2015 | 10 | 1 | — | — |  |  |  |  |  |  |  |  |  |  |  |
| 2016 | 129 | 24 | 0.85 | 0.10, 7.31 |  |  |  |  |  |  |  |  |  |  |  |
| 2017 | 4,599 | 176 | 0.24 | 0.03, 1.95 |  |  |  |  |  |  |  |  |  |  |  |
| 2018 | 1,895 | 42 | 0.16 | 0.02, 1.32 |  |  |  |  |  |  |  |  |  |  |  |
| 2019 | 2,412 | 88 | 0.19 | 0.02, 1.58 |  |  |  |  |  |  |  |  |  |  |  |
| 2020 |  |  |  |  |  | 998 | 22 | — | — | 0.006 |  |  |  |  |  |
| 2021 |  |  |  |  |  | 1,493 | 13 | 0.44 | 0.22, 0.88 |  |  |  |  |  |  |
| 2022 |  |  |  |  |  | 2,876 | 71 | 1.14 | 0.69, 1.88 |  |  |  |  |  |  |
| 2023 |  |  |  |  |  |  |  |  |  |  | 2,742 | 107 | — | — | 0.5 |
| 2024 |  |  |  |  |  |  |  |  |  |  | 4,182 | 184 | 0.91 | 0.70, 1.18 |  |
| **Geographic region** | |  |  |  | <0.001 |  |  |  |  | <0.001 |  |  |  |  | 0.2 |
| North | 1,251 | 7 | — | — |  | 280 | 0 | — | — |  | 231 | 2 | — | — |  |
| Northeast | 4,524 | 210 | 6.34 | 2.96, 13.6 |  | 3,935 | 83 | 17,149 | 12,870, 22,850 | | 2,014 | 50 | 2.22 | 0.53, 9.22 |  |
| Southeast | 2,960 | 105 | 5.7 | 2.63, 12.3 |  | 848 | 14 | 16,959 | 9,697, 29,660 | | 3,586 | 194 | 2.92 | 0.71, 11.9 |  |
| South | 36 | 1 | 4.68 | 0.54, 40.9 |  | 31 | 0 | 1.64 | 0.92, 2.93 |  | 152 | 4 | 1.44 | 0.26, 7.85 |  |
| Central west | 274 | 8 | 6.06 | 2.17, 16.9 |  | 273 | 9 | 34,458 | 17,572, 67,572 | | 941 | 41 | 2.69 | 0.64, 11.3 |  |
| **Sex** |  |  |  |  | 0.001 |  |  |  |  | 0.041 |  |  |  |  | <0.001 |
| Female | 5,278 | 162 | — | — |  | 3,032 | 49 | — | — |  | 3,796 | 130 | — | — |  |
| Male | 3,767 | 169 | 1.43 | 1.15, 1.78 |  | 2,335 | 57 | 1.5 | 1.02, 2.22 |  | 3,128 | 161 | 1.63 | 1.28, 2.06 |  |
| **Age group** |  |  |  |  | <0.001 |  |  |  |  | <0.001 |  |  |  |  | <0.001 |
| 20-39 | 2,362 | 31 | — | — |  | 1,367 | 22 | — | — |  | 1,511 | 32 | — | — |  |
| <1 | 433 | 21 | 3.5 | 1.99, 6.17 |  | 268 | 8 | 1.85 | 0.80, 4.32 |  | 336 | 9 | 1.28 | 0.60, 2.75 |  |
| 1-9 | 1,204 | 10 | 0.62 | 0.30, 1.26 |  | 858 | 5 | 0.34 | 0.13, 0.90 |  | 988 | 5 | 0.24 | 0.09, 0.63 |  |
| 10-19 | 1,267 | 17 | 0.99 | 0.55, 1.80 |  | 642 | 9 | 0.88 | 0.40, 1.92 |  | 770 | 9 | 0.55 | 0.26, 1.17 |  |
| 40-49 | 1,043 | 24 | 1.71 | 1.00, 2.92 |  | 677 | 10 | 0.92 | 0.44, 1.94 |  | 692 | 18 | 1.08 | 0.60, 1.94 |  |
| 50-59 | 976 | 28 | 2 | 1.19, 3.36 |  | 542 | 6 | 0.65 | 0.27, 1.60 |  | 707 | 29 | 1.39 | 0.82, 2.34 |  |
| 60-69 | 791 | 39 | 3.16 | 1.94, 5.13 |  | 435 | 8 | 0.97 | 0.42, 2.23 |  | 700 | 36 | 1.55 | 0.95, 2.54 |  |
| 70-79 | 549 | 61 | 6.53 | 4.15, 10.3 |  | 335 | 19 | 2.74 | 1.39, 5.42 |  | 664 | 65 | 2.83 | 1.78, 4.49 |  |
| 80-89 | 352 | 78 | 13.8 | 8.95, 21.3 |  | 192 | 13 | 3 | 1.43, 6.31 |  | 453 | 67 | 4.39 | 2.76, 6.98 |  |
| ≥90 | 68 | 22 | 16.4 | 8.87, 30.3 |  | 51 | 6 | 4.43 | 1.53, 12.8 |  | 103 | 21 | 8.31 | 4.72, 14.6 |  |
| **Diabetes** |  |  |  |  | <0.001 |  |  |  |  | 0.2 |  |  |  |  | <0.001 |
| No | 8,460 | 255 | — | — |  | 4,952 | 85 | — | — |  | 6,194 | 197 | — | — |  |
| Yes | 585 | 76 | 1.81 | 1.34, 2.45 |  | 415 | 21 | 1.55 | 0.84, 2.86 |  | 730 | 94 | 1.83 | 1.38, 2.44 |  |
| **Autoimmune disease** | |  |  |  | 0.12 |  |  |  |  | 0.5 |  |  |  |  | 0.2 |
| No | 8,954 | 326 | — | — |  | 5,300 | 103 | — | — |  | 6,778 | 279 | — | — |  |
| Yes | 91 | 5 | 2.01 | 0.83, 4.86 |  | 67 | 3 | 1.73 | 0.37, 8.18 |  | 146 | 12 | 1.57 | 0.80, 3.05 |  |
| **Hematologic disease** | |  |  |  | 0.2 |  |  |  |  | 0.6 |  |  |  |  | 0.2 |
| No | 8,972 | 329 | — | — |  | 5,306 | 104 | — | — |  | 6,831 | 287 | — | — |  |
| Yes | 73 | 2 | 0.36 | 0.07, 1.91 |  | 61 | 2 | 0.58 | 0.08, 4.48 |  | 93 | 4 | 0.38 | 0.10, 1.42 |  |
| **Liver disease** | |  |  |  | >0.9 |  |  |  |  | 0.2 |  |  |  |  | <0.001 |
| No | 8,957 | 327 | — | — |  | 5,286 | 101 | — | — |  | 6,835 | 274 | — | — |  |
| Yes | 88 | 4 | 0.96 | 0.30, 3.06 |  | 81 | 5 | 2.07 | 0.73, 5.88 |  | 89 | 17 | 4.51 | 2.51, 8.10 |  |
| **Hypertension** | |  |  |  | 0.6 |  |  |  |  | 0.8 |  |  |  |  | 0.012 |
| No | 7,718 | 208 | — | — |  | 4,536 | 76 | — | — |  | 5,519 | 140 | — | — |  |
| Yes | 1,327 | 123 | 1.07 | 0.81, 1.41 |  | 831 | 30 | 0.93 | 0.52, 1.67 |  | 1,405 | 151 | 1.46 | 1.09, 1.97 |  |
| **Kidney disease** | |  |  |  | 0.041 |  |  |  |  | 0.001 |  |  |  |  | 0.016 |
| No | 8,925 | 309 | — | — |  | 5,285 | 95 | — | — |  | 6,773 | 267 | — | — |  |
| Yes | 120 | 22 | 1.64 | 1.02, 2.63 |  | 82 | 11 | 3.33 | 1.62, 6.84 |  | 151 | 24 | 1.73 | 1.11, 2.71 |  |

**Death: Other causes**

|  | **Pre** | | | | | **During** | | | | | **Post** | | | | |
| --- | --- | --- | --- | --- | --- | --- | --- | --- | --- | --- | --- | --- | --- | --- | --- |
| **Characteristic** | **N** | **Event N** | **sHR** | **95% CI** | **p-value** | **N** | **Event N** | **sHR** | **95% CI** | **p-value** | **N** | **Event N** | **sHR** | **95% CI** | **p-value** |
| **Year** |  |  |  |  | <0.001 |  |  |  |  |  |  |  |  |  |  |
| 2015 | 10 | 0 | — | — |  | — | — | — | — |  | — | — | — | — |  |
| 2016 | 129 | 1 | 2,556 | 310, 21,100 |  | — | — | — | — |  | — | — | — | — |  |
| 2017 | 4,599 | 42 | 3,809 | 1,614, 8,991 |  | — | — | — | — |  | — | — | — | — |  |
| 2018 | 1,895 | 18 | 4,611 | 1,810, 11,742 | | — | — | — | — |  | — | — | — | — |  |
| 2019 | 2,412 | 22 | 4,236 | 1,722, 10,421 | | — | — | — | — | 0.003 | — | — | — | — |  |
| 2020 | — | — | — | — |  | 998 | 27 | — | — |  | — | — | — | — |  |
| 2021 | — | — | — | — |  | 1,493 | 23 | 0.56 | 0.32, 0.97 |  | — | — | — | — |  |
| 2022 | — | — | — | — |  | 2,876 | 26 | 0.36 | 0.20, 0.66 |  | — | — | — | — | 0.8 |
| 2023 | — | — | — | — |  |  |  |  |  |  | 2,742 | 64 | — | — |  |
| 2024 | — | — | — | — |  |  |  |  |  |  | 4,182 | 93 | 0.94 | 0.64, 1.39 |  |
| **Geographic region** | |  |  |  | <0.001 |  |  |  |  | <0.001 |  |  |  |  | 0.035 |
| North | 1,251 | 9 | — | — |  | 280 | 6 | — | — |  | 231 | 9 | — | — |  |
| Northeast | 4,524 | 49 | 1.38 | 0.66, 2.87 |  | 3,935 | 42 | 0.38 | 0.16, 0.91 |  | 2,014 | 42 | 0.47 | 0.23, 0.98 |  |
| Southeast | 2,960 | 20 | 0.77 | 0.33, 1.80 |  | 848 | 23 | 0.84 | 0.32, 2.19 |  | 3,586 | 74 | 0.35 | 0.17, 0.73 |  |
| South | 36 | 0 | 0 | 0.00, 0.00 |  | 31 | 0 | 0 | 0.00, 0.00 |  | 152 | 5 | 0.59 | 0.21, 1.68 |  |
| Central west | 274 | 5 | 2.14 | 0.69, 6.59 |  | 273 | 5 | 0.79 | 0.24, 2.62 |  | 941 | 27 | 0.54 | 0.24, 1.20 |  |
| **Sex** |  |  |  |  | 0.4 |  |  |  |  | 0.2 |  |  |  |  | 0.083 |
| Female | 5,278 | 47 | — | — |  | 3,032 | 37 | — | — |  | 3,796 | 77 | — | — |  |
| Male | 3,767 | 36 | 1.2 | 0.76, 1.91 |  | 2,335 | 39 | 1.37 | 0.85, 2.21 |  | 3,128 | 80 | 1.33 | 0.96, 1.83 |  |
| **Age group** |  |  |  |  | 0.11 |  |  |  |  | 0.069 |  |  |  |  | 0.002 |
| 20-39 | 2,362 | 18 | — | — |  | 1,367 | 12 | — | — |  | 1,511 | 25 | — | — |  |
| <1 | 433 | 6 | 1.76 | 0.68, 4.57 |  | 268 | 5 | 2.16 | 0.74, 6.29 |  | 336 | 8 | 1.37 | 0.61, 3.06 |  |
| 1-9 | 1,204 | 5 | 0.52 | 0.19, 1.42 |  | 858 | 10 | 1.28 | 0.54, 3.05 |  | 988 | 11 | 0.62 | 0.30, 1.28 |  |
| 10-19 | 1,267 | 6 | 0.58 | 0.22, 1.52 |  | 642 | 6 | 1.07 | 0.40, 2.90 |  | 770 | 13 | 0.99 | 0.51, 1.95 |  |
| 40-49 | 1,043 | 12 | 1.43 | 0.68, 2.98 |  | 677 | 7 | 1.03 | 0.41, 2.60 |  | 692 | 14 | 1.26 | 0.65, 2.43 |  |
| 50-59 | 976 | 15 | 1.86 | 0.93, 3.73 |  | 542 | 10 | 1.79 | 0.74, 4.33 |  | 707 | 14 | 1.15 | 0.59, 2.22 |  |
| 60-69 | 791 | 10 | 1.35 | 0.62, 2.95 |  | 435 | 9 | 1.83 | 0.70, 4.74 |  | 700 | 24 | 1.85 | 1.02, 3.35 |  |
| 70-79 | 549 | 4 | 0.7 | 0.21, 2.30 |  | 335 | 4 | 0.98 | 0.30, 3.18 |  | 664 | 18 | 1.51 | 0.79, 2.88 |  |
| 80-89 | 352 | 5 | 1.39 | 0.51, 3.79 |  | 192 | 10 | 4.36 | 1.60, 11.9 |  | 453 | 22 | 2.85 | 1.47, 5.54 |  |
| ≥90 | 68 | 2 | 3.02 | 0.69, 13.3 |  | 51 | 3 | 5.58 | 1.42, 21.9 |  | 103 | 8 | 4.84 | 2.10, 11.2 |  |
| **Diabetes** |  |  |  |  | <0.001 |  |  |  |  | 0.6 |  |  |  |  | >0.9 |
| No | 8,460 | 66 | — | — |  | 4,952 | 64 | — | — |  | 6,194 | 130 | — | — |  |
| Yes | 585 | 17 | 3.21 | 1.66, 6.19 |  | 415 | 12 | 1.23 | 0.61, 2.47 |  | 730 | 27 | 1.02 | 0.62, 1.68 |  |
| **Autoimmune disease** | |  |  |  | 0.11 |  |  |  |  | 0.026 |  |  |  |  | 0.3 |
| No | 8,954 | 80 | — | — |  | 5,300 | 73 | — | — |  | 6,778 | 149 | — | — |  |
| Yes | 91 | 3 | 3.46 | 0.74, 16.2 |  | 67 | 3 | 3.65 | 1.17, 11.4 |  | 146 | 8 | 1.69 | 0.64, 4.44 |  |
| **Hematologic disease** | |  |  |  | 0.07 |  |  |  |  | <0.001 |  |  |  |  | 0.12 |
| No | 8,972 | 80 | — | — |  | 5,306 | 76 | — | — |  | 6,831 | 150 | — | — |  |
| Yes | 73 | 3 | 4.36 | 0.89, 21.4 |  | 61 | 0 | 0 | 0.00, 0.00 |  | 93 | 7 | 2.24 | 0.81, 6.20 |  |
| **Liver disease** | |  |  |  | 0.4 |  |  |  |  | 0.2 |  |  |  |  | 0.2 |
| No | 8,957 | 82 | — | — |  | 5,286 | 73 | — | — |  | 6,835 | 150 | — | — |  |
| Yes | 88 | 1 | 0.26 | 0.01, 6.08 |  | 81 | 3 | 2.08 | 0.61, 7.05 |  | 89 | 7 | 2.01 | 0.71, 5.74 |  |
| **Hypertension** | |  |  |  | 0.7 |  |  |  |  | 0.14 |  |  |  |  | 0.3 |
| No | 7,718 | 63 | — | — |  | 4,536 | 52 | — | — |  | 5,519 | 103 | — | — |  |
| Yes | 1,327 | 20 | 0.9 | 0.51, 1.58 |  | 831 | 24 | 1.72 | 0.84, 3.52 |  | 1,405 | 54 | 1.29 | 0.82, 2.03 |  |
| **Kidney disease** | |  |  |  | >0.9 |  |  |  |  | 0.6 |  |  |  |  | 0.2 |
| No | 8,925 | 80 | — | — |  | 5,285 | 73 | — | — |  | 6,773 | 147 | — | — |  |
| Yes | 120 | 3 | 1.09 | 0.24, 4.90 |  | 82 | 3 | 1.41 | 0.39, 5.04 |  | 151 | 10 | 1.69 | 0.77, 3.73 |  |

# Supplementary Table 10: Risk factors for death following dengue estimated using Fine and Gray model stratified by period of COVID-19 pandemic. Pre-COVID (2015-2019), During COVID-19 (2020-2022) and Post COVID-19 (2023-2024). sHR = sub distribution hazard ratio; CI = Confidence Interval

**Death: Dengue**

|  | **Pre** | | | | | **During** | | | | | **Post** | | | | |
| --- | --- | --- | --- | --- | --- | --- | --- | --- | --- | --- | --- | --- | --- | --- | --- |
| **Characteristic** | **N** | **Event N** | **HR** | **95% CI** | **p-value** | **N** | **Event N** | **HR** | **95% CI** | **p-value** | **N** | **Event N** | **HR** | **95% CI** | **p-value** |
| **Year** |  |  |  |  | <0.001 |  |  |  |  |  |  |  |  |  |  |
| 2015 | 36,576 | 747 | — | — |  | — | — | — | — |  | — | — | — | — |  |
| 2016 | 28,725 | 601 | 0.93 | 0.84, 1.04 |  | — | — | — | — |  | — | — | — | — |  |
| 2017 | 10,068 | 148 | 0.72 | 0.60, 0.86 |  | — | — | — | — |  | — | — | — | — |  |
| 2018 | 13,309 | 174 | 0.58 | 0.49, 0.69 |  | — | — | — | — |  | — | — | — | — |  |
| 2019 | 50,871 | 707 | 0.46 | 0.41, 0.52 |  | — | — | — | — | 0.014 | — | — | — | — |  |
| 2020 | — | — | — | — |  | 28,836 | 478 | — | — |  | — | — | — | — |  |
| 2021 | — | — | — | — |  | 15,359 | 225 | 1.13 | 0.96, 1.33 |  | — | — | — | — |  |
| 2022 | — | — | — | — |  | 47,348 | 882 | 1.18 | 1.06, 1.32 |  | — | — | — | — | <0.001 |
| 2023 | — | — | — | — |  | — | — | — | — |  | 46,636 | 936 | — | — |  |
| 2024 | — | — | — | — |  | — | — | — | — |  | 178,129 | 5,087 | 1.24 | 1.15, 1.33 |  |
| **Geographic region** | |  |  |  | <0.001 |  |  |  |  | <0.001 |  |  |  |  | <0.001 |
| North | 7,343 | 47 | — | — |  | 6,350 | 75 | — | — |  | 4,747 | 60 | — | — |  |
| Northeast | 29,663 | 359 | 1.87 | 1.38, 2.54 |  | 16,795 | 189 | 0.91 | 0.69, 1.19 |  | 20,854 | 294 | 1.08 | 0.82, 1.43 |  |
| Southeast | 56,009 | 1,368 | 2.54 | 1.90, 3.41 |  | 21,001 | 525 | 1.31 | 1.03, 1.68 |  | 110,718 | 3,387 | 1.41 | 1.09, 1.82 |  |
| South | 8,352 | 102 | 1.28 | 0.90, 1.80 |  | 17,533 | 433 | 1.22 | 0.95, 1.56 |  | 49,877 | 1,362 | 1.23 | 0.95, 1.59 |  |
| Central west | 38,182 | 501 | 1.65 | 1.22, 2.24 |  | 29,864 | 363 | 0.76 | 0.59, 0.98 |  | 38,569 | 920 | 1.3 | 1.00, 1.69 |  |
| **Sex** |  |  |  |  | <0.001 |  |  |  |  | 0.001 |  |  |  |  | <0.001 |
| Female | 75,379 | 1,135 | — | — |  | 48,673 | 793 | — | — |  | 121,603 | 3,026 | — | — |  |
| Male | 64,170 | 1,242 | 1.34 | 1.24, 1.46 |  | 42,870 | 792 | 1.18 | 1.07, 1.30 |  | 103,162 | 2,997 | 1.23 | 1.17, 1.29 |  |
| **Age group** |  |  |  |  | <0.001 |  |  |  |  | <0.001 |  |  |  |  | <0.001 |
| 20-39 | 40,274 | 364 | — | — |  | 24,107 | 212 | — | — |  | 54,079 | 687 | — | — |  |
| <1 | 3,249 | 22 | 0.74 | 0.48, 1.14 |  | 2,084 | 25 | 1.45 | 0.95, 2.19 |  | 4,060 | 62 | 1.2 | 0.93, 1.56 |  |
| 1-9 | 14,585 | 85 | 0.66 | 0.52, 0.84 |  | 10,828 | 49 | 0.55 | 0.40, 0.75 |  | 21,538 | 89 | 0.33 | 0.26, 0.41 |  |
| 10-19 | 21,369 | 125 | 0.65 | 0.53, 0.79 |  | 13,222 | 74 | 0.66 | 0.50, 0.86 |  | 28,856 | 169 | 0.46 | 0.39, 0.55 |  |
| 40-49 | 17,636 | 263 | 1.56 | 1.33, 1.83 |  | 11,243 | 149 | 1.38 | 1.12, 1.70 |  | 27,055 | 488 | 1.31 | 1.17, 1.48 |  |
| 50-59 | 16,352 | 333 | 1.93 | 1.66, 2.24 |  | 10,287 | 185 | 1.66 | 1.36, 2.03 |  | 24,794 | 626 | 1.64 | 1.47, 1.83 |  |
| 60-69 | 12,465 | 346 | 2.37 | 2.04, 2.75 |  | 8,615 | 225 | 2.14 | 1.77, 2.60 |  | 24,842 | 917 | 2.18 | 1.96, 2.41 |  |
| 70-79 | 8,565 | 381 | 3.49 | 3.01, 4.05 |  | 6,666 | 298 | 3.38 | 2.80, 4.09 |  | 22,303 | 1,276 | 3.15 | 2.86, 3.48 |  |
| 80-89 | 4,281 | 348 | 6.26 | 5.38, 7.29 |  | 3,713 | 273 | 5.56 | 4.59, 6.74 |  | 13,988 | 1,241 | 4.87 | 4.40, 5.38 |  |
| ≥90 | 773 | 110 | 11.4 | 9.17, 14.0 |  | 778 | 95 | 9.9 | 7.69, 12.7 |  | 3,250 | 468 | 8.42 | 7.45, 9.52 |  |
| **Diabetes** |  |  |  |  | <0.001 |  |  |  |  | <0.001 |  |  |  |  | <0.001 |
| No | 134,532 | 2,067 | — | — |  | 84,616 | 1,216 | — | — |  | 202,298 | 4,445 | — | — |  |
| Yes | 5,017 | 310 | 1.55 | 1.32, 1.83 |  | 6,927 | 369 | 1.43 | 1.25, 1.64 |  | 22,467 | 1,578 | 1.34 | 1.25, 1.43 |  |
| **Autoimmune disease** | |  |  |  | 0.15 |  |  |  |  | 0.3 |  |  |  |  | <0.001 |
| No | 138,711 | 2,329 | — | — |  | 90,461 | 1,535 | — | — |  | 221,135 | 5,813 | — | — |  |
| Yes | 838 | 48 | 1.41 | 0.89, 2.25 |  | 1,082 | 50 | 1.23 | 0.84, 1.81 |  | 3,630 | 210 | 1.38 | 1.17, 1.63 |  |
| **Hematologic disease** | |  |  |  | 0.3 |  |  |  |  | <0.001 |  |  |  |  | <0.001 |
| No | 138,632 | 2,326 | — | — |  | 90,489 | 1,524 | — | — |  | 221,730 | 5,781 | — | — |  |
| Yes | 917 | 51 | 1.32 | 0.82, 2.10 |  | 1,054 | 61 | 1.92 | 1.37, 2.70 |  | 3,035 | 242 | 2.12 | 1.80, 2.48 |  |
| **Liver disease** | |  |  |  | 0.03 |  |  |  |  | 0.032 |  |  |  |  | 0.6 |
| No | 138,562 | 2,316 | — | — |  | 90,466 | 1,526 | — | — |  | 222,293 | 5,886 | — | — |  |
| Yes | 987 | 61 | 1.53 | 1.04, 2.25 |  | 1,077 | 59 | 1.48 | 1.04, 2.12 |  | 2,472 | 137 | 0.95 | 0.76, 1.18 |  |
| **Hypertension** | |  |  |  | <0.001 |  |  |  |  | <0.001 |  |  |  |  | <0.001 |
| No | 128,819 | 1,788 | — | — |  | 76,799 | 893 | — | — |  | 179,533 | 3,114 | — | — |  |
| Yes | 10,730 | 589 | 2.2 | 1.92, 2.53 |  | 14,744 | 692 | 1.53 | 1.35, 1.73 |  | 45,232 | 2,909 | 1.51 | 1.42, 1.60 |  |
| **Kidney disease** | |  |  |  | <0.001 |  |  |  |  | <0.001 |  |  |  |  | <0.001 |
| No | 138,496 | 2,273 | — | — |  | 90,268 | 1,468 | — | — |  | 220,668 | 5,542 | — | — |  |
| Yes | 1,053 | 104 | 2.07 | 1.60, 2.68 |  | 1,275 | 117 | 2.23 | 1.77, 2.81 |  | 4,097 | 481 | 2.14 | 1.93, 2.37 |  |

**Death: Other causes**

|  | **Pre** | | | | | **During** | | | | | **Post** | | | | |
| --- | --- | --- | --- | --- | --- | --- | --- | --- | --- | --- | --- | --- | --- | --- | --- |
| **Characteristic** | **N** | **Event N** | **HR** | **95% CI** | **p-value** | **N** | **Event N** | **HR** | **95% CI** | **p-value** | **N** | **Event N** | **HR** | **95% CI** | **p-value** |
| **Year** |  |  |  |  | <0.001 |  |  |  |  |  |  |  |  |  |  |
| 2015 | 36,576 | 181 | — | — |  | — | — | — | — |  | — | — | — | — |  |
| 2016 | 28,725 | 207 | 1.33 | 1.09, 1.62 |  | — | — | — | — |  | — | — | — | — |  |
| 2017 | 10,068 | 89 | 1.82 | 1.39, 2.38 |  | — | — | — | — |  | — | — | — | — |  |
| 2018 | 13,309 | 65 | 0.99 | 0.73, 1.33 |  | — | — | — | — |  | — | — | — | — |  |
| 2019 | 50,871 | 203 | 0.61 | 0.49, 0.76 |  | — | — | — | — | <0.001 | — | — | — | — |  |
| 2020 | — | — | — | — |  | 28,836 | 222 | — | — |  | — | — | — | — |  |
| 2021 | — | — | — | — |  | 15,359 | 140 | 1.33 | 1.07, 1.65 |  | — | — | — | — |  |
| 2022 | — | — | — | — |  | 47,348 | 338 | 0.91 | 0.77, 1.08 |  | — | — | — | — | 0.11 |
| 2023 | — | — | — | — |  | — | — | — | — |  | 46,636 | 305 | — | — |  |
| 2024 | — | — | — | — |  | — | — | — | — |  | 178,129 | 1,230 | 0.9 | 0.80, 1.02 |  |
| **Geographic region** | |  |  |  | <0.001 |  |  |  |  | <0.001 |  |  |  |  | <0.001 |
| North | 7,343 | 39 | — | — |  | 6,350 | 46 | — | — |  | 4,747 | 33 | — | — |  |
| Northeast | 29,663 | 157 | 1.08 | 0.76, 1.54 |  | 16,795 | 96 | 0.8 | 0.56, 1.14 |  | 20,854 | 138 | 0.97 | 0.66, 1.41 |  |
| Southeast | 56,009 | 396 | 1.12 | 0.80, 1.57 |  | 21,001 | 222 | 0.87 | 0.63, 1.21 |  | 110,718 | 940 | 0.72 | 0.51, 1.03 |  |
| South | 8,352 | 38 | 0.72 | 0.46, 1.14 |  | 17,533 | 109 | 0.49 | 0.34, 0.69 |  | 49,877 | 280 | 0.46 | 0.32, 0.66 |  |
| Central west | 38,182 | 115 | 0.51 | 0.35, 0.74 |  | 29,864 | 227 | 0.78 | 0.56, 1.07 |  | 38,569 | 144 | 0.37 | 0.25, 0.55 |  |
| **Sex** |  |  |  |  | <0.001 |  |  |  |  | <0.001 |  |  |  |  | <0.001 |
| Female | 75,379 | 321 | — | — |  | 48,673 | 285 | — | — |  | 121,603 | 713 | — | — |  |
| Male | 64,170 | 424 | 1.6 | 1.38, 1.85 |  | 42,870 | 415 | 1.69 | 1.46, 1.97 |  | 103,162 | 822 | 1.4 | 1.27, 1.55 |  |
| **Age group** |  |  |  |  | <0.001 |  |  |  |  | <0.001 |  |  |  |  | <0.001 |
| 20-39 | 40,274 | 135 | — | — |  | 24,107 | 95 | — | — |  | 54,079 | 138 | — | — |  |
| <1 | 3,249 | 13 | 1.08 | 0.61, 1.90 |  | 2,084 | 12 | 1.33 | 0.73, 2.43 |  | 4,060 | 23 | 2.12 | 1.36, 3.31 |  |
| 1-9 | 14,585 | 41 | 0.76 | 0.54, 1.09 |  | 10,828 | 19 | 0.42 | 0.25, 0.68 |  | 21,538 | 21 | 0.36 | 0.23, 0.57 |  |
| 10-19 | 21,369 | 43 | 0.57 | 0.40, 0.80 |  | 13,222 | 14 | 0.25 | 0.14, 0.44 |  | 28,856 | 41 | 0.51 | 0.36, 0.73 |  |
| 40-49 | 17,636 | 98 | 1.63 | 1.26, 2.12 |  | 11,243 | 54 | 1.17 | 0.84, 1.63 |  | 27,055 | 109 | 1.53 | 1.19, 1.97 |  |
| 50-59 | 16,352 | 87 | 1.43 | 1.09, 1.88 |  | 10,287 | 92 | 2.07 | 1.55, 2.76 |  | 24,794 | 183 | 2.63 | 2.10, 3.29 |  |
| 60-69 | 12,465 | 108 | 2.18 | 1.68, 2.82 |  | 8,615 | 123 | 3.17 | 2.40, 4.18 |  | 24,842 | 257 | 3.48 | 2.81, 4.30 |  |
| 70-79 | 8,565 | 103 | 2.81 | 2.15, 3.67 |  | 6,666 | 130 | 4.28 | 3.22, 5.69 |  | 22,303 | 299 | 4.29 | 3.47, 5.32 |  |
| 80-89 | 4,281 | 97 | 5.15 | 3.94, 6.75 |  | 3,713 | 124 | 7.43 | 5.58, 9.91 |  | 13,988 | 337 | 7.72 | 6.23, 9.56 |  |
| ≥90 | 773 | 20 | 5.86 | 3.63, 9.47 |  | 778 | 37 | 11.2 | 7.52, 16.6 |  | 3,250 | 127 | 12.9 | 10.0, 16.6 |  |
| **Diabetes** |  |  |  |  | <0.001 |  |  |  |  | 0.017 |  |  |  |  | 0.026 |
| No | 134,532 | 644 | — | — |  | 84,616 | 550 | — | — |  | 202,298 | 1,167 | — | — |  |
| Yes | 5,017 | 101 | 1.94 | 1.45, 2.60 |  | 6,927 | 150 | 1.29 | 1.05, 1.59 |  | 22,467 | 368 | 1.17 | 1.02, 1.33 |  |
| **Autoimmune disease** | |  |  |  | 0.5 |  |  |  |  | 0.012 |  |  |  |  | 0.008 |
| No | 138,711 | 727 | — | — |  | 90,461 | 671 | — | — |  | 221,135 | 1,477 | — | — |  |
| Yes | 838 | 18 | 1.3 | 0.58, 2.91 |  | 1,082 | 29 | 1.87 | 1.15, 3.04 |  | 3,630 | 58 | 1.53 | 1.12, 2.09 |  |
| **Hematologic disease** | |  |  |  | >0.9 |  |  |  |  | 0.2 |  |  |  |  | 0.019 |
| No | 138,632 | 727 | — | — |  | 90,489 | 675 | — | — |  | 221,730 | 1,483 | — | — |  |
| Yes | 917 | 18 | 1 | 0.41, 2.45 |  | 1,054 | 25 | 1.46 | 0.83, 2.56 |  | 3,035 | 52 | 1.53 | 1.07, 2.17 |  |
| **Liver disease** | |  |  |  | <0.001 |  |  |  |  | 0.002 |  |  |  |  | 0.037 |
| No | 138,562 | 711 | — | — |  | 90,466 | 666 | — | — |  | 222,293 | 1,487 | — | — |  |
| Yes | 987 | 34 | 3.35 | 1.93, 5.83 |  | 1,077 | 34 | 2.16 | 1.34, 3.48 |  | 2,472 | 48 | 1.47 | 1.02, 2.12 |  |
| **Hypertension** | |  |  |  | 0.002 |  |  |  |  | 0.053 |  |  |  |  | <0.001 |
| No | 128,819 | 588 | — | — |  | 76,799 | 424 | — | — |  | 179,533 | 830 | — | — |  |
| Yes | 10,730 | 157 | 1.5 | 1.16, 1.92 |  | 14,744 | 276 | 1.2 | 1.00, 1.44 |  | 45,232 | 705 | 1.3 | 1.15, 1.47 |  |
| **Kidney disease** | |  |  |  | 0.029 |  |  |  |  | <0.001 |  |  |  |  | <0.001 |
| No | 138,496 | 712 | — | — |  | 90,268 | 650 | — | — |  | 220,668 | 1,398 | — | — |  |
| Yes | 1,053 | 33 | 1.84 | 1.06, 3.19 |  | 1,275 | 50 | 2 | 1.40, 2.86 |  | 4,097 | 137 | 2.38 | 1.95, 2.91 |  |

# Supplementary Table 11: Number of deaths by proportion of minorities by municipality

|  |  |  | **Chikungunya** | | | **Dengue** | |
| --- | --- | --- | --- | --- | --- | --- | --- |
| **Quintile** | **% of minorities** | **Population** | | **Number of Deaths** | **Average age of Death** | **Number of Deaths** | **Average age of Death** |
|  |  |  | |  |  |  |  |
| 1 - Highest | ≥78.7 | 26400503 (13%) | | 112 | 46.8 | 563 | 43.9 |
| 2 | 68.5-78.6 | 42424885 (21%) | | 320 | 51.7 | 1585 | 50.1 |
| 3 | 53.4-68.4 | 51848998 (25%) | | 505 | 58.0 | 3378 | 57.4 |
| 4 | 33.5-53.3 | 48515146 (24%) | | 91 | 61.4 | 4764 | 65.1 |
| 5 - Lowest | 2.5-33.4 | 33891224 (17%) | | 16 | 69.6 | 2670 | 67.7 |

**Quintile calculated of the proportion of Black, mixed and indigenous population in each municipality.**

***The SII/RII in the article was calculated using the individual level data**


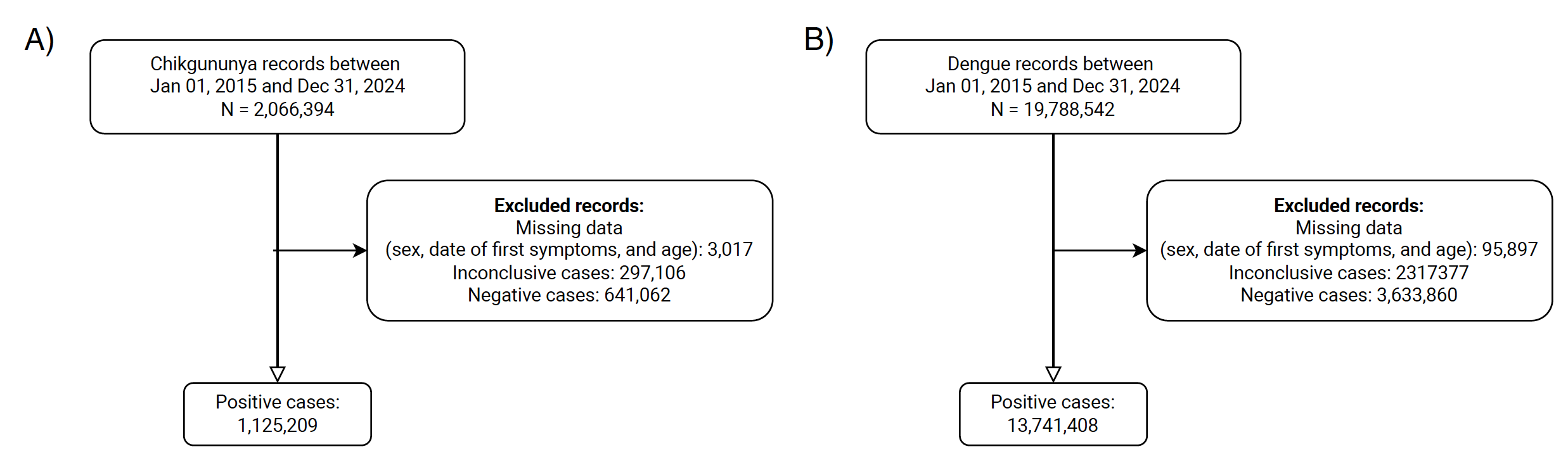


# Supplementary Figure 1: Flowchart of the study population from surveillance databases


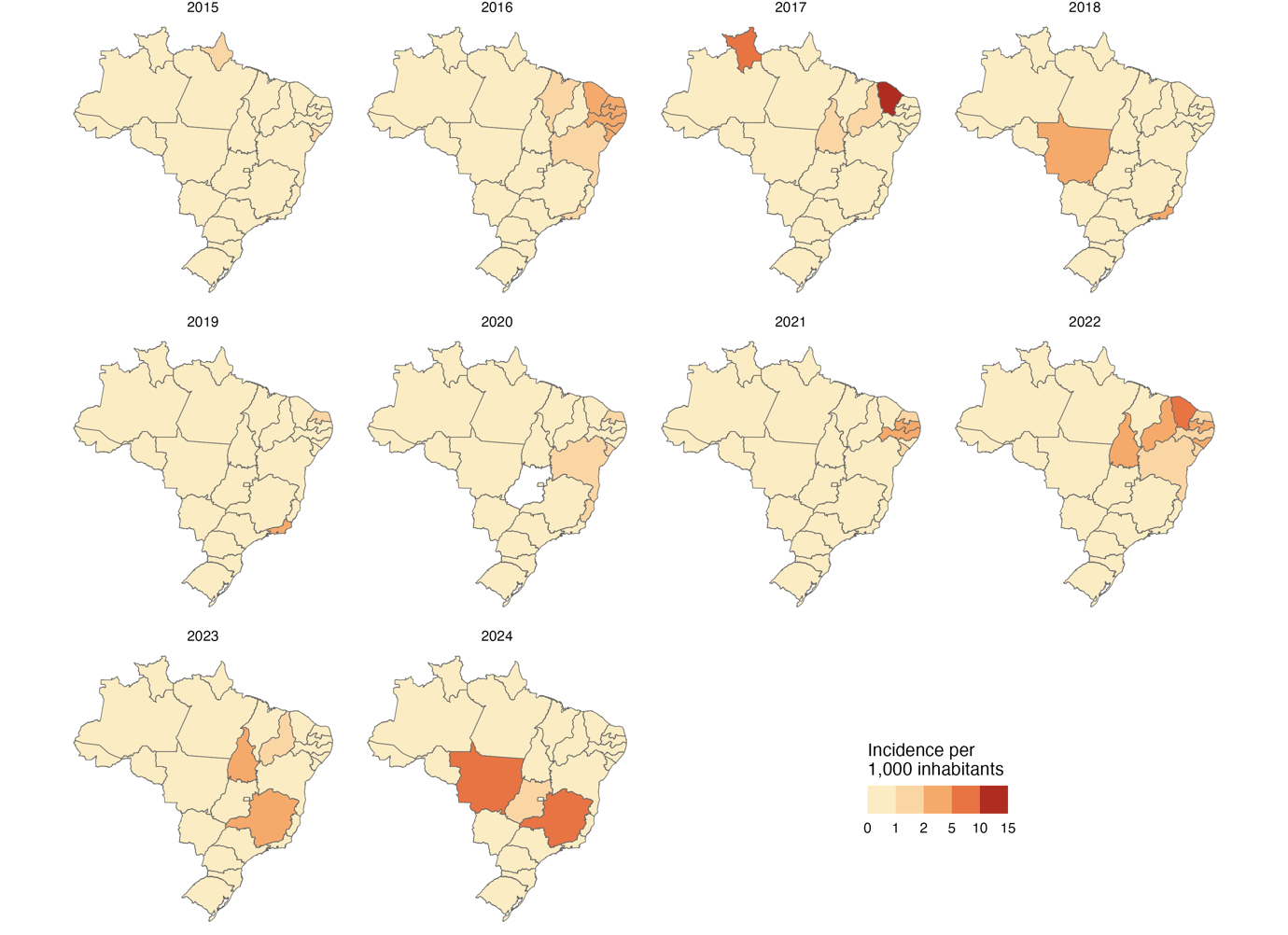


# Supplementary Figure 2: Incidence per year of chikungunya by State in Brazil.


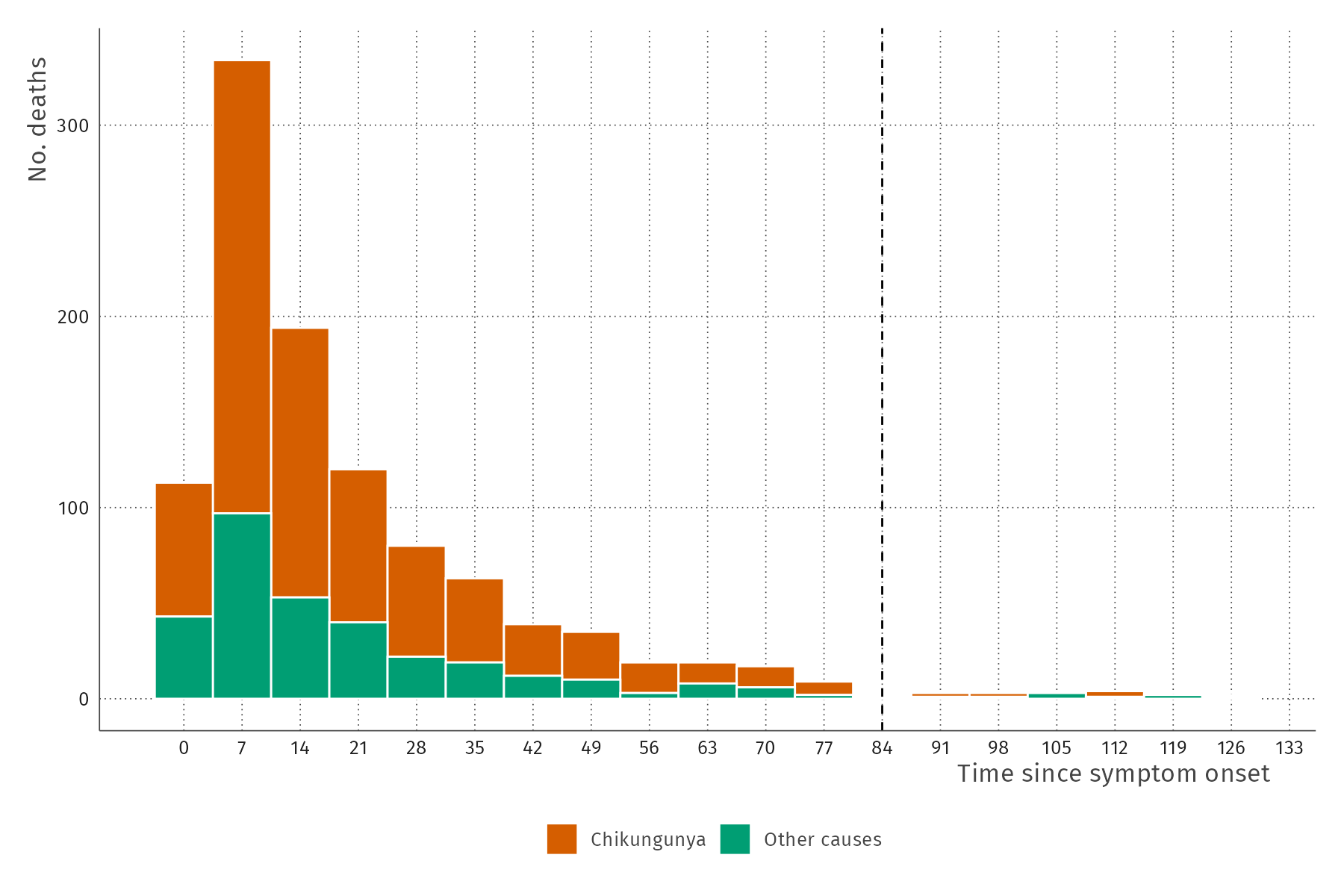


# Supplementary Figure 3: Histogram with the number of in-patient deaths after chikungunya by time since symptom onset stratified by the cause of death


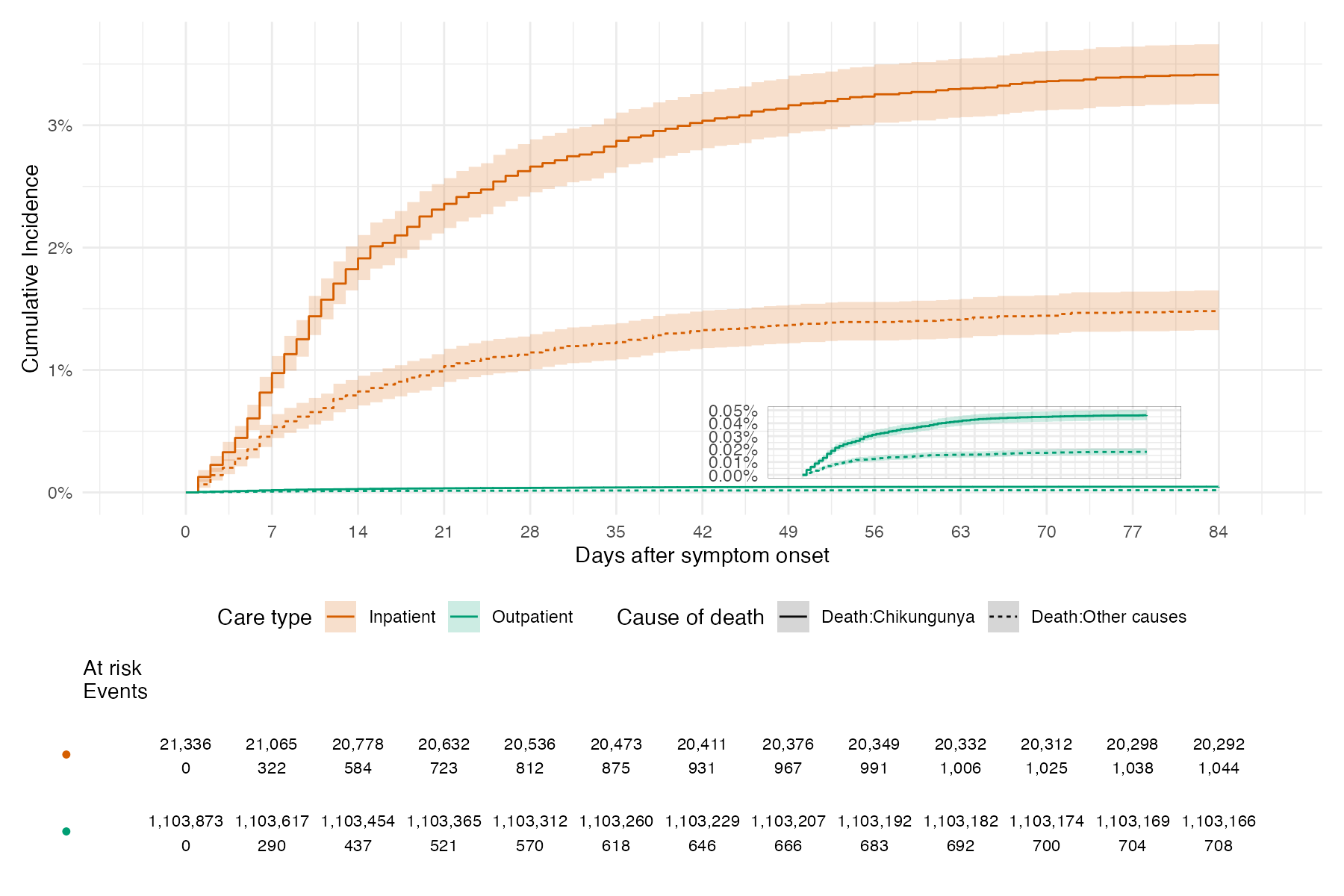


# Supplementary Figure 4: Cumulative incidence function of death after chikungunya by time since symptom onset.


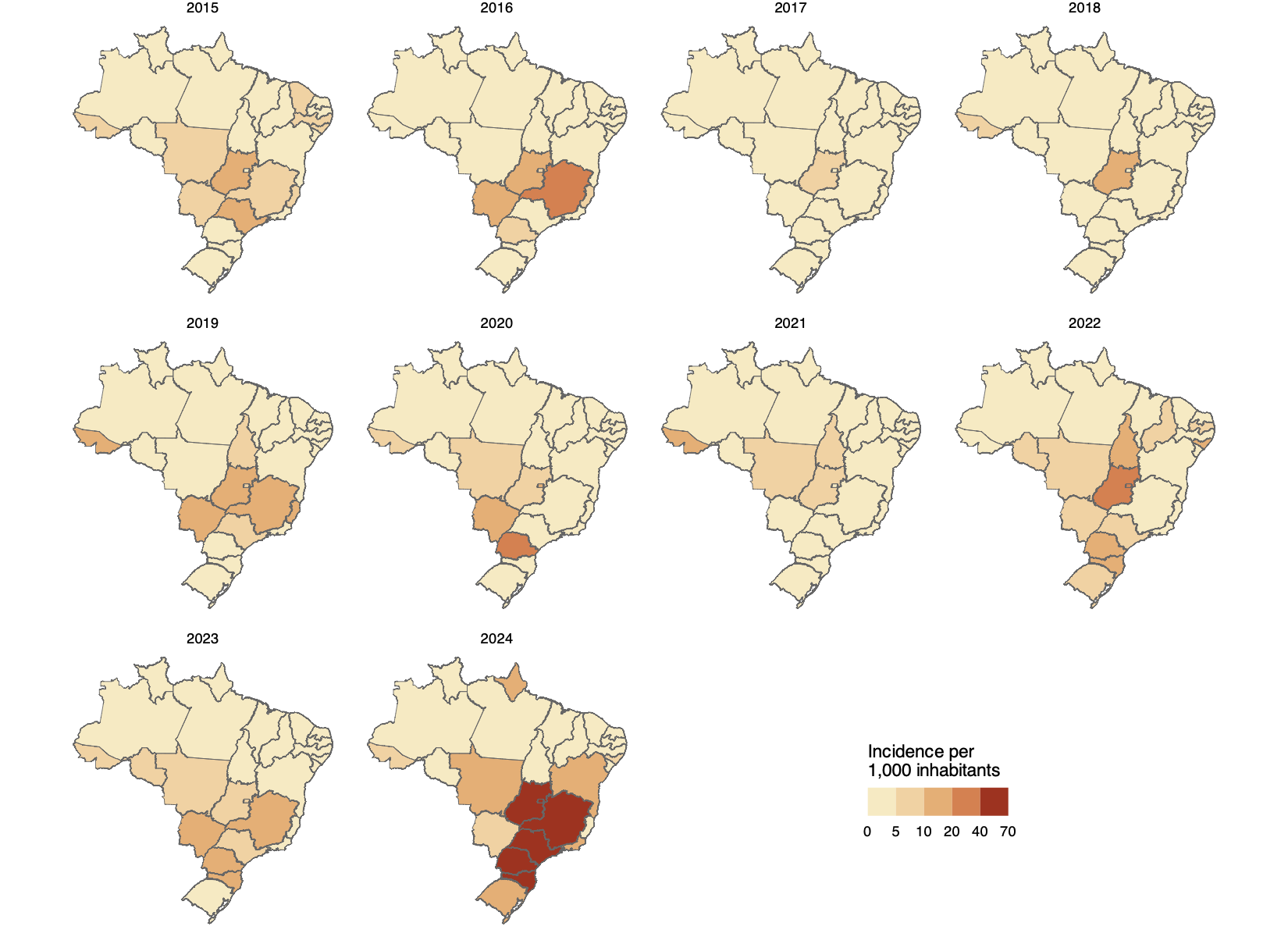


# Supplementary Figure 5: Incidence per year of dengue by State in Brazil.


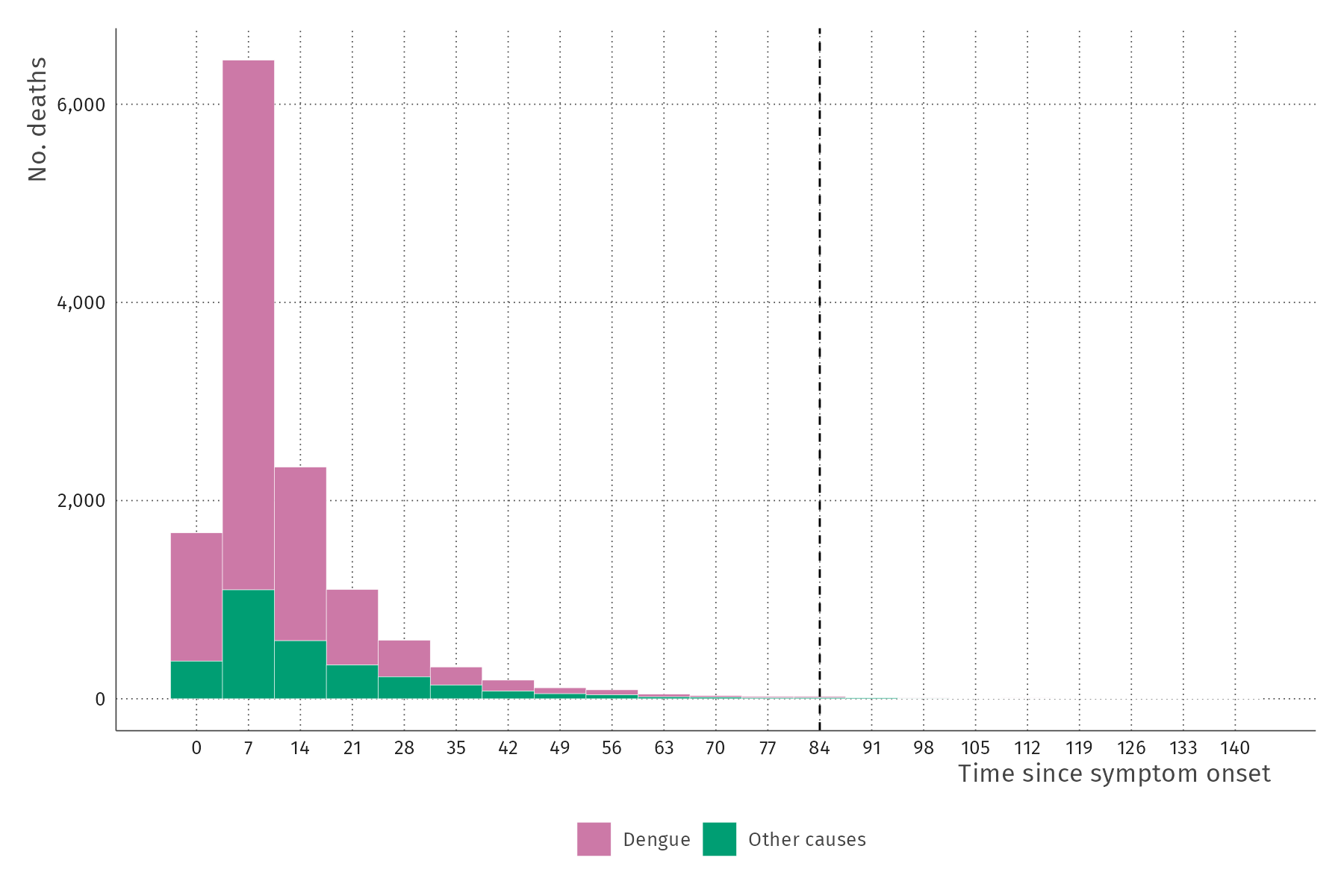


# Supplementary Figure 6: Histogram with the number of in-patient deaths after dengue by time since symptom onset stratified by the cause of death


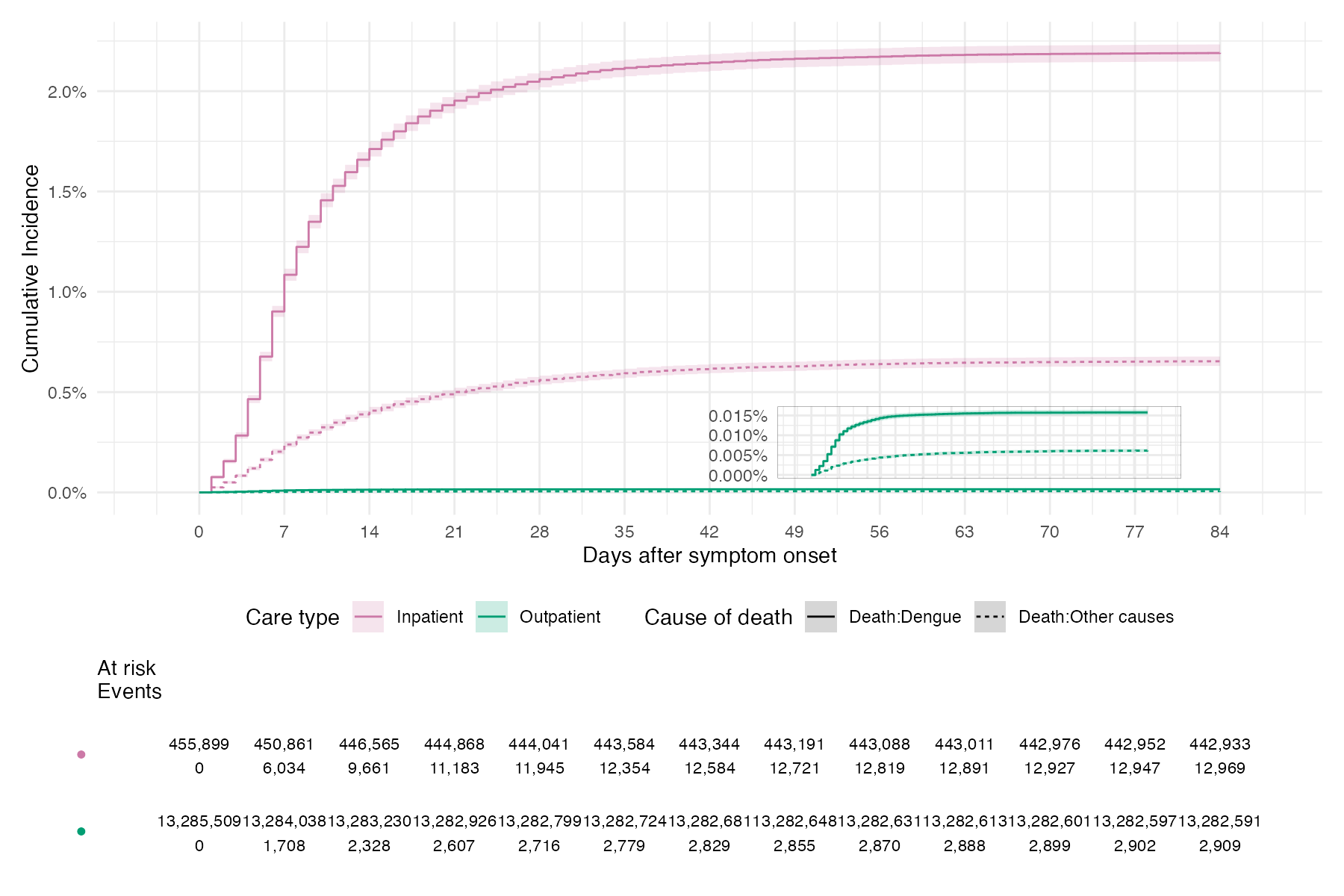


# Supplementary Figure 7: Cumulative incidence function of death after dengue by time since symptom onset.

# Appendix Methods

The **cumulative incidence function (CIF)** for event type k can be defined as:

$$I_{k}\left( t \right) = P\left( T\leq t, D=k \right)$$

In the presence of competing risks, the cause-specific hazards for cause k can be defined as:


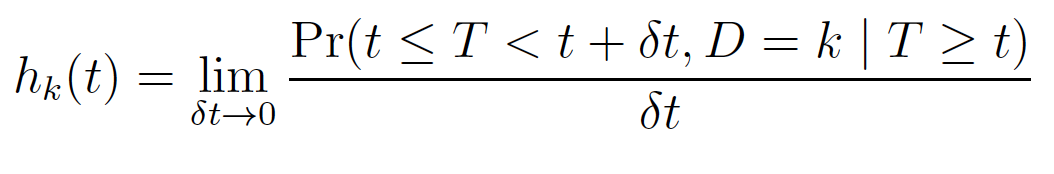
 or


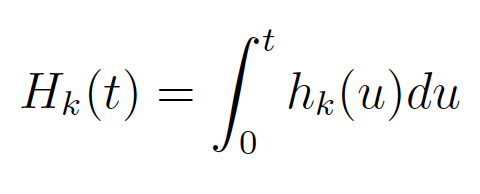


The overall hazard of death is the sum of all-cause specific hazards:


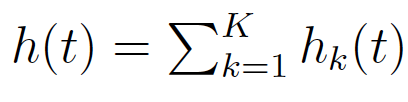


And the overall survival function can be defined as:


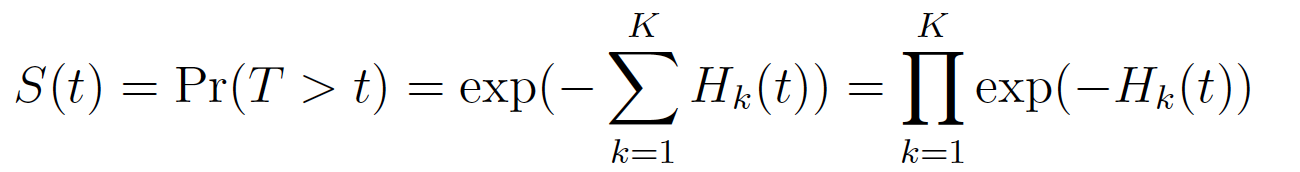


This way, the cumulative incidence function can be defined as:


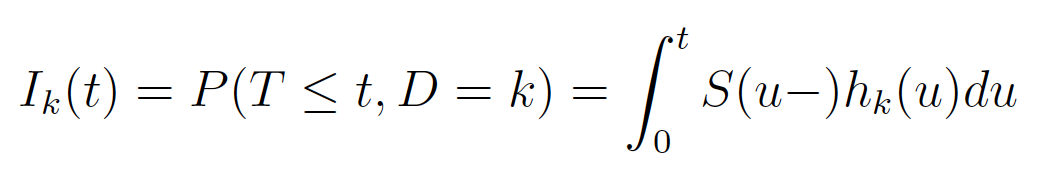


Where $S\left( u- \right)$ is the overall survival just before time *u* and $h_{k}\left( u \right)$ is the cause-specific hazard.

The regression to estimate the cumulative incidence in the context of subdistribution hazard can be obtained through the **Fine and Gray** model as:


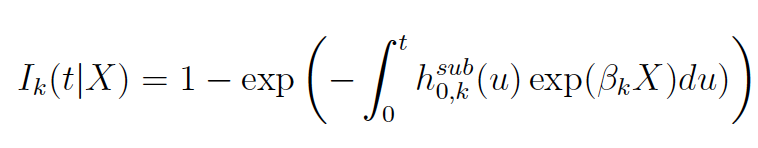


**Years of life lost (YLL)**

The equations for estimating YLL and aYLL are:


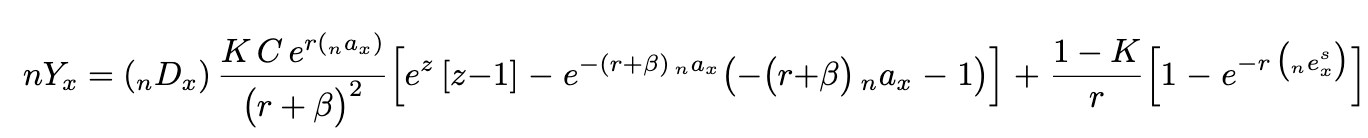

$$\text{YLL}=\sum{{}_{n}Y}_{x}$$

$$aYLL=\frac{YLL}{N}$$

The parameters are:

$x$: Age
$n:$ Age interval length
$e_{x}$:Standard life expectancy at age x
${}_{n}e_{x}$: Standard life expectancy for age interval x to x + n
${}_{x}a_{x}$: Average age of death for age interval x to x + n
${{}_{n}Y}_{x}$: Expected years of life lost for age interval x to x + n
${}_{n}D_{x}$: Number of deaths in age interval x to x + n
*r*: Discount rate (usually set to 0.03)
K: Age-weighting modulation constant (K = 0, no weighting; K = 1, weighting)
$\beta$: Age-weighting constant (set to = 0.04)
C: Adjustment constant for age-weights (set to 0.1658)
*N*: total number of deaths

In this study, age weighting was not used (K=0), and the discount rate used was 0.03 (r=0.03)
