# Supplementary material for: Hospitalisation, mortality and years of life lost among chikungunya and dengue cases in Brazil: a nationwide cohort study, 2015–2024
Source: Lancet Reg Health Am. 2025 Jul 7;49:101177. doi: 10.1016/j.lana.2025.101177 (PMC12273570; doi:10.1016/j.lana.2025.101177)
Supplement: Translated Summary [file mmc2.pdf]

Editorial Disclaimer: *This translation in Portuguese was submitted by the authors and we reproduce it as supplied. It has not been peer reviewed. Our editorial processes have only been applied to the original abstract in English, which should serve as a reference for this manuscript.*

Resumo

Contexto: A incidência de infecções por vírus transmitidos por artrópodes, incluindo chikungunya e dengue, está aumentando globalmente. Utilizamos dados nacionais coletados ao longo de uma década no Brasil para examinar os fatores associados à hospitalização, mortalidade hospitalar e os anos de vida perdidos por essas doenças no país.

Métodos: Utilizando registros nacionais anonimizados de chikungunya e dengue notificados entre 1º de janeiro de 2015 e 31 de dezembro de 2024, estimamos os fatores de risco para hospitalização e mortalidade hospitalar por meio de regressão logística e do modelo de Fine e Gray, respectivamente. Também calculamos os anos de vida perdidos (*Years of Life Lost* - YLL) para cada doença e a média de anos de vida perdidos (*average Years of Life Lost* -aYLL), estratificados por região geográfica, sexo e raça/cor.

Resultados: Foram analisados 1.125.209 casos de chikungunya: 21.336 (1,9%) necessitaram de hospitalização. Entre os hospitalizados, ocorreram 1.044 (4,9%) óbitos até 84 dias do início dos sintomas, dos quais 728 (69,7%) foram atribuídos à chikungunya. Foram analisados 13.741.408 casos de dengue: 455.899 (3,3%) necessitaram de hospitalização, com 12.969 (2,8%) óbitos entre os hospitalizados, sendo 9.989 (77,0%) atribuídos à dengue. Idade (<1 ou ≥70 anos), sexo masculino e presença de diabetes e doença renal foram fatores de risco para hospitalização e mortalidade hospitalar em ambas as doenças. O aYLL para chikungunya foi de 16,0 anos, e para dengue, 14,5 anos; no entanto, o impacto não foi distribuído de forma uniformemente na população. Para chikungunya, participantes autodeclarados pretos apresentaram o maior aYLL (22,0 anos), enquanto os brancos foram os menos afetados (aYLL: 13,0). Para dengue, o grupo mais afetado foi o indígena (aYLL: 22,5) e o menos afetado, o branco (aYLL: 12,6).

Interpretação: Bebês (<1 ano), idosos (≥70 anos), sexo masculino e presença de comorbidades estão associados a maior gravidade nos casos de chikungunya e dengue. Essas doenças afetam desproporcionalmente populações historicamente minorizadas, com participantes autodeclarados pretos e indígenas apresentando significativamente mais anos de vida perdidos em comparação com a população branca. Para mitigar os impactos da chikungunya e da dengue é necessário enfrentar as desigualdades sociais e de saúde.

Financiamento: Royal Society, Wellcome Trust, CNPq
